# Applying the algorithm for Proven and young in GWAS Reveals high polygenicity for key traits in Nellore cattle

**DOI:** 10.3389/fgene.2025.1549284

**Published:** 2025-04-30

**Authors:** Adebisi R. Ogunbawo, Jorge Hidalgo, Henrique A. Mulim, Eula R. Carrara, Henrique T. Ventura, Nadson O. Souza, Daniela Lourenco, Hinayah R. Oliveira

**Affiliations:** ^1^ Department of Animal Sciences, Purdue University, West Lafayette, IN, United States; ^2^ Department of Animal and Dairy Sciences, University of Georgia, Athens, GA, United States; ^3^ Brazilian Association of Zebu Breeders, Uberaba, Minas Gerais, Brazil

**Keywords:** candidate genes, genomic regions, genomic estimated breeding value, GWAS, single step

## Abstract

**Background:**

Identifying genomic regions associated with traits of interest and their biological processes provides valuable insights into the phenotypic variability of these traits. This study aimed to identify candidate genes and genomic regions associated with 16 traits currently evaluated by the Brazilian Association of Zebu Breeders (ABCZ). These traits include reproductive traits such as age at first calving (AFC), stayability (STAY), and scrotal circumference at 365 (SC365) and 450 days (SC450). Growth traits include birthweight (BW), expected progeny difference for weight at 120days of age (EPD120), as well as weight at 120 (W120), 210 (W210), 365 (W365), and 450 days of age (W450). Carcass traits include body conformation (BC), finishing score (FS), marbling (MARB), muscularity (MUSC), finishing precocity (FP), and ribeye area (REA).

**Methods:**

A dataset containing 304,782 Nellore cattle genotyped with 437,650 SNPs (after quality control) was used for this study. The Algorithm for Proven and Young (APY), implemented in the PREGSF90 software, was used to compute the $G_{APY}^{-1}$ matrix using 36,000 core animals (which explained 98% of the variance in the genomic matrix). Subsequently, the SNP solutions were estimated by back-solving the Genomic Estimated Breeding Values (GEBVs) predicted by ABCZ using the single-step GBLUP method. Genomic regions were identified using sliding windows of 175 consecutive SNPs, and the top 1% genomic windows, ranked based on their proportion of the additive genetic variance, were used to annotate positional candidate genes and genomic regions associated with each of the 16 traits.

**Results:**

The top 1% windows for all traits explained between 2.779% (STAY) to 3.158% (FP) of the additive genetic variance, highlighting the polygenic nature of these traits. Functional analysis of the candidate genes and genomic regions provided valuable insights into the genetic architecture underlying these traits in Nellore cattle. For instance, our results revealed genes with important functions for each trait, such as *SERPINA14* (plays a key role for the endometrial epithelium) identified for AFC, *HSPG2* (associated with morphological development and tissue differentiation) identified for BW, among others.

**Conclusion:**

We identified genomic regions and candidate genes, some of which have been previously reported in the literature, while others are novel discoveries that warrant further investigation. These findings contribute to gene prioritization efforts, facilitating the identification of functional candidate genes that can enhance genomic selection strategies for economically important traits in Nellore cattle.

## 1 Introduction

Genome-wide association studies (GWAS) are paramount for identifying genetic variants associated with complex traits in livestock, thereby enhancing the accuracy of genomic predictions (Sahana et al., 2023). By leveraging high-throughput genotyping, GWAS enables the identification of candidate genes and genomic regions associated with traits of interest in livestock by exploiting the non-random association of alleles in the cattle genome (Mkize et al., 2021; Wang et al., 2005). Many studies using small genomic datasets for GWAS tend to report either fewer associations or a high number of potentially spurious ones, particularly if adequate multiple-test correction methods are not properly implemented (Galliou et al., 2020). In contrast, research based on larger datasets typically identifies a higher number of more reliable associations due to their increased statistical power (Jiang et al., 2019), making them less susceptible to the identification of false positives (Misztal et al., 2021).

Nellore cattle, a *Bos taurus indicus* breed, play a vital role in the global beef industry, representing over 80% of Brazil’s beef cattle population and solidifying Brazil’s position as one of the world’s largest beef exporters (Josahkian, 2000; USDA, 2011; 2019; Carvalheiro et al., 2014). Various breeding programs focusing on genomic selection aim to accelerate genetic progress, reduce generational interval, and increase the accuracy of selection in this breed (e.g., Carvalheiro, 2014; Albuquerque et al., 2018; Fernandes Júnior et al., 2022). In this context, previous studies have investigated genomic regions associated with key traits in Nellore cattle, such as age at puberty in young bulls using 18,746 genotyped animals (Stafuzza et al., 2020); carcass quality using 502 genotyped animals (Carvalho et al., 2020a); visual score trait using 2,775 genotyped animals (Machado et al., 2022), and scrotal circumference using 3,450 genotyped animals (Irano et al., 2016). Additionally, Schmidt et al. (2023) used GWAS to identify significant SNP markers located within genomic regions harboring lethal haplotypes associated with heifer rebreeding, post-natal mortality, and stayability in a population of 62,022 genotyped animals. However, to the best of our knowledge, no GWAS to date have yet used datasets exceeding 65,000 genotyped Nellore animals. Performing GWAS using larger datasets could substantially enhance the power to detect candidate genes associated with economically important traits in Nellore cattle, avoiding the spurious associations of small studies (Pocrnic et al., 2024).

The Brazilian Association of Zebu Breeders (ABCZ; Uberaba, MG, Brazil) is a prominent cattle association that manages the largest Zebu database in Brazil, with over 12 million registered animals nationwide. The ABCZ monitors the genetic improvement of more than 3,600 herds across the country, performing official genetic and genomic evaluations for seven different dairy and beef cattle Zebu breeds. The Nellore breeding program, established in the 1950s, is one of the largest breeding programs for this breed in the world, with over 300,000 genotyped animals and more than 14 million animals in the pedigree (includes registered and non-registered animals from commercial herds).

In the single-step genomic evaluations based on the Genomic Best Linear Unbiased Prediction (GBLUP) approach, the most computationally intensive step is the inversion of the genomic relationship matrix (**G**), which is later combined with the inverse of the pedigree-based relationship matrix (**A**) to build the inverse of the **H** matrix (Aguilar et al., 2010). For large genotyped populations, directly inverting **G** becomes computationally infeasible, as the complexity of these operations scales cubically with the number of genotypes (Fragomeni et al., 2015a). However, with advanced computational algorithms, these operations have been shown to be possible for datasets with up to ∼2.3 million genotyped individuals (Fragomeni et al., 2015b; Masuda, 2019).

The Algorithm for Proven and Young (APY) allows for the efficient implementation of large genotyped populations by separating the genotyped animals into core and non-core groups; the core group carries information about the independent chromosome segments segregating in the population and is the portion of **G** that demands direct inversion; however, it has limited dimensionality, allowing an efficient inversion of **G** (Misztal et al., 2014a; Misztal, 2015; Fernando et al., 2016; Pocrnic et al., 2016a; Bradford et al., 2017). This method enables large-scale genomic evaluations, allowing breeding programs to leverage the growing amount of genomic data to accelerate genetic gain and optimize selection process (Bradford et al., 2017; Doublet et al., 2019; Leite et al., 2024). While APY has been widely used for genomic predictions (e.g., Misztal et al., 2020), limited research has used APY in GWAS (Leite et al., 2024; Aguilar et al., 2019; Misztal et al., 2024). Consequently, our objective in this study is to perform GWAS using one of the world’s largest genotyped populations of Nellore cattle, leveraging the APY algorithm to identify candidate genes and genomic regions associated with the 16 traits currently evaluated by ABCZ.

## 2 Materials and methods

Animal Care and Use Committee approval was not needed for this study, as all data were obtained from an existing database.

### 2.1 Datasets

The Brazilian Association of Zebu Breeders (ABCZ; https://www.abcz.org.br) provided the variance components, pedigree, genotypes, and genomic estimated breeding values (GEBVs) from their December 2023 official genomic evaluation of Nellore cattle. This dataset includes GEBVs for 304,782 Nellore animals across the 16 traits currently analyzed in their national evaluation. The reproductive traits include age at first calving (AFC), stayability (STAY), and scrotal circumference at 365 (SC365) and 450 days (SC450). Growth traits include birthweight (BW), expected progeny difference for weight at 120days of age (EPD120), as well as weight at 120 (W120), 210 (W210), 365 (W365), and 450 days of age (W450). Carcass traits include body conformation (BC), finishing score (FS), marbling (MARB), muscularity (MUSC), finishing precocity (FP), and ribeye area (REA) (Rizzo et al., 2015; Costa et al., 2020). Details about the statistical models currently used by ABCZ to evaluate the mentioned traits and quality control performed in the phenotypes are available at the ABCZ website (https://www.abczstat.com.br/comunicacoes/sumario/apresentacao/Sumario-racas-NEL.htm). In summary, the GEBVs were predicted using the single-step Genomic Best Linear Unbiased Prediction (ssGBLUP) method (Legarra et al., 2009; Aguilar et al., 2010), using linear animal mixed models for all traits (including visual score traits) except STAY (for STAY a threshold model was used). The statistical model used for STAY incorporated only the fixed effect of contemporary group and the additive genetic random effect. The statistical model used for AFC incorporated the fixed effects of contemporary group and age of the dam (in classes), and the additive genetic random effect. For all other traits evaluated in this study, the statistical models included the fixed effects of contemporary group, animal age at the measurement (as a covariable, nested in the contemporary group), age of the dam (in classes), and the additive random effect. The covariable age at the measurement is routinely used by ABCZ in their official genetic evaluations to further correct for any residual age-related variation that may persist despite the standard age adjustments. For the genomic evaluation of weights recorded before weaning (i.e., BW, W120, and W210), the statistical model additionally included maternal genetic and permanent maternal environmental effects.

Pedigree information was available for approximately 14 million Nellore animals raised in Brazil. Out of these, 309,640 animals were genotyped using 14 commercially available single nucleotide polymorphisms (SNP) panels, which ranged from low to high-density. After defining the optimal imputation approach for this population, imputation was performed using the FImpute v3 (Sargolzaei et al., 2014) software, considering pedigree information. The imputation was performed in two steps: first, all low and medium-density genotypes were imputed to a custom SNP panel containing approximately 120 k SNPs, which was created by combining the 50 k and 70 k panels (over 86 k animals in the reference). Subsequently, the 120 k SNP panel was imputed to the high-density SNP panel (777 k), using 1,962 animals genotyped with high-density SNP panel in the reference. Quality control performed before imputation kept only SNPs mapped in the autosomes with call rate above 0.90, minor allele frequencies above 0.01, and deviation from the Hardy–Weinberg equilibrium lower than 0.15. Imputation accuracy was greater than 0.98 for the dataset used.

### 2.2 Genotypic quality control

Genotypic quality control was performed using the QCF90 software from the BLUPF90 family of programs (Misztal et al., 2002). After quality control, 179,575 SNPs were excluded based on minor allele frequency (MAF) < 0.05, extreme deviation from Hardy-Weinberg equilibrium (>0.15; calculated as the difference between observed and expected heterozygote frequencies; following Wiggans et al., 2009), and SNP markers located on non-autosomal regions. Finally, a total of 304,782 animals and 437,650 SNPs distributed across the 29 autosomal chromosomes remained for further analysis.

### 2.3 Statistical analyses

#### 2.3.1 Algorithm for proven and young

To facilitate obtaining the inverse of the genetic relationship matrix (**G**), the Algorithm for Proven and Young (APY) was used in the analysis (Misztal et al., 2014a; Mrode and Pocrnic, 2023). The number of core animals in APY was identified using the PREGSF90 program (Misztal et al., 2014b; Lourenco et al., 2022), which assesses the number of eigenvalues of **G** that explains the largest variance. For computational reasons, the eigenvalues are obtained by squaring the singular values; therefore, PREGSF90 performs the Singular Value Decomposition (SVD) of the SNP content matrix, **Z** (Pocrnic et al., 2016b). Pocrnic et al. (2016b) demonstrated that the number of core animals can be determined by the number of eigenvalues of G that explain 98%–99% of the variance in this matrix.

In this study, we found that about 36,000 core animals captured 98% of the variance in **G**, therefore, we randomly selected 36,000 core animals to ensure a representative sample of the population, as suggested by Pocrnic et al. (2016a) and Garcia et al. (2022). The relationships in **G** ^-1^ APY ( $G_{\text{APY}}^{-1}$ ) were then computed through recursions based on the core set, with linear computation cost for noncore and cubic for core animals. The inverse of the genomic relationship matrix with APY was constructed as (Pocrnic et al., 2019; Leite et al., 2024):

$$G_{\text{APY}}^{-1}=\left[\begin{array}{cc} G_{\text{cc}}^{-1} & 0\\ 0 & 0 \end{array}\right]+\left[\begin{array}{c} {-G}_{cc}^{-1}G_{cn}\\ I \end{array}\right]M_{\text{nn}}^{-1}\left[{-G}_{\text{nc}}G_{\text{cc}}^{-1}\;I\right]$$

where $G_{\text{APY}}^{-1}$ is the inverse of the genomic relationship matrix with APY, $G_{\text{cc}}^{-1}$ and $M_{\text{nn}}^{-1}$ are the inverses of the genomic relationship matrix for core and diagonal for noncore animals respectively. The $G_{\text{cn}}$ is the genomic relationship matrix between core and noncore animals*,* $G_{\text{nc}}$ is the genomic relationship matrix between noncore and core animals, and **I** is the identity matrix. The $M_{\text{nn}}^{-1}$ is a diagonal matrix, with its element derived as: $M_{\text{nn}}=\text{diag}\left\{m_{\text{nn},j}\right\}=\text{diag}\left\{g_{\text{jj}}-g_{\text{jc}}^{\prime }\;G_{\text{cc}}^{-1}g_{\text{cj}}\right\},$ where $g_{\text{jj}}$ is the diagonal element of $G_{\text{nn}}$ for the $j^{\text{th}}$ animal, and $g_{\text{jc}}$ is the relationship between the $j^{\text{th}}$ noncore animal with core animals. Using APY, the computational cost in inverting a dense **G** is reduced to only inverting the genomic relationship matrix for core animals (i.e., $G_{\text{cc}}),$ which is sparse (Pocrnic et al., 2019; Leite et al., 2024).

#### 2.3.2 Genome-wide association studies

The POSTGSF90 program (Misztal et al., 2014b; Lourenco et al., 2022) was used to obtain the SNP effects by back-solving the genomic estimated breeding values (GEBVs) for each trait using the following equation (VanRaden, 2008; Strandén and Garrick, 2009; Wang et al., 2012):

$$\hat{a}=\left(1-\beta \right)\;b\frac{\sigma_{u}^{2}}{\sigma_{a}^{2}}\;Z^{\prime }G_{APY}^{-1}\hat{u},$$

where $\hat{a}$ is the vector of estimated SNP effects, β is the blending parameter (5%) to avoid singularity problems in **G** (VanRaden, 2008 **),** b is a tuning parameter (Vitezica et al., 2011), $\sigma_{u}^{2}$ is SNP variance, $\sigma_{a}^{2}$ is the genetic variance, Z is a matrix of SNP content centered by two times the allele frequency (p), $\hat{u}$ is the vector of the GEBVs provided by ABCZ, and $G_{\text{APY}}^{-1}$ is the inverse of the genomic relationship matrix constructed using APY.

To identify important genomic regions associated with the analyzed traits, we determined the percentage of variance explained by moving windows of 175 adjacent SNPs. This window size was chosen based on Pocrnic et al. (2024), who analytically proved that 80% of QTL variance can be captured by eight Stam segments. Following Pocrnic et al. (2024), the mean length of a Stam segment (in Morgans) can be computed as $\frac{1}{4Ne}$ ; where Ne is the effective population size, which for this population is to 196; therefore, the mean length of a Stam segment would be 0.00128 Morgans, and 8 Stam segments span over 0.0102 Morgans or 1.02 Mb. Thus, our windows size contained 175 SNPs (using our SNP panel after quality control). This approach balances the need to capture genetic variance with minimizing noise from excessively large or small window sizes (Fragomeni et al., 2014). The additive genetic variance explained by the *i*th SNP ( $\sigma_{a\left(i\right)}^{2}$ ) was estimated as follows (Fragomeni et al., 2014; Zhang et al., 2010):

$$\sigma_{a\left(i\right)}^{2}={\hat{a}}_{i}^{2}2p_{i}\left(1-p_{i}\right),$$

where ${\hat{a}}_{i}^{2}$ is the estimated effect of *k*th SNP for the analyzed trait, and all other terms were previously defined. The total additive genetic variance explained by each genomic window was calculated by summing the variance explained by the 175 consecutive SNPs. Only windows explaining the highest proportion of variance for each trait were retained for further analysis. The top 1% windows for each trait (i.e., n = 20 windows per trait) were used for gene annotation in order to retrieve important genomic regions for all analyzed traits.

#### 2.3.3 Genome annotation and functional enrichment analyses

The top 1% windows for each trait were used for the annotation of genes located within the windows. Gene annotation was performed using the GALLO package (Fonseca et al., 2020) available in the R software (R Core Team, 2024), based on the Ensembl database (http://useast.ensembl.org/index.html; accessed October 2024) using the ARS-UCD1.2 genome assembly (Rosen et al., 2020). Additionally, the gprofiler2 package (Kolberg et al., 2020) available in R (R Core Team, 2024) was used to perform the functional analysis according to the similarity of the biological process, molecular functions, cellular components, and Kyoto Encyclopedia of Genes and Genomes (KEGG) pathways associated with the identified candidate genes with p-value <0.05 were stated as significant.

## 3 Results

### 3.1 Genome windows and identification of candidate genes

**FIGURE 1:**
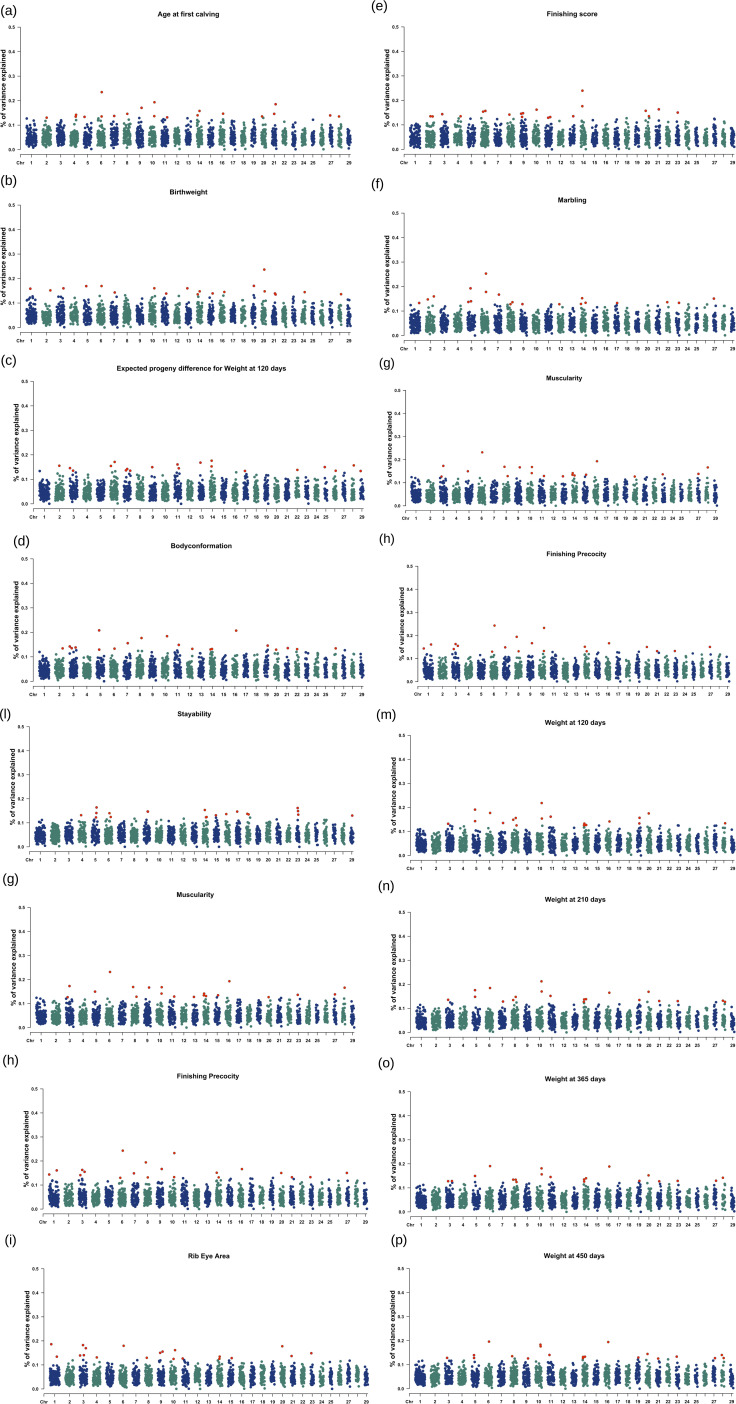
**(a–p)** Manhattan plot showing the top 1% windows with the highest proportion of genetic variance explained across chromosomes. The red dot represents the top 1% region associated with trait of interest. The Y axis represents “% of variance” explained for each chromosome.

Manhattan plots showing the top 1% genomic windows explaining the highest proportion of the additive genetic variance are shown in Figure 1. The top 1% genomic windows and candidate genes highlighted for all 16 traits are detailed in the sections below.

#### 3.1.1 Age at first calving

The top 1% genomic window were located across 15 chromosomes as seen in Figure 1a (i.e., BTA2, BTA4, BTA5, BTA6, BTA7, BTA8, BTA9, BTA10, BTA11, BTA14, BTA16, BTA20, BTA21, BTA27, BTA28), explaining a minimum additive genetic variance of 0.130% at BTA2 and maximum additive genetic variance of 0.235% at BTA6. Collectively, these windows explained 3.010% of the total additive genetic variance for AFC. The top 1% windows, along with their genomic positions, overlapped 225 positional candidate genes, classified as protein-coding (n = 144), long noncoding RNAs (n = 63), small noncoding RNAs (n = 9), and miscellaneous noncoding RNAs (n = 4), as detailed in Supplementary Table S1A. In summary, nine molecular functions which are statistically associated with the genes and related to age at first calving in Nellore are highlighted in the Supplementary Table S1B.

#### 3.1.2 Birth weight

The top 1% genomic window were located across 17 chromosomes as seen in Figure 1b (i.e., BTA1, BTA2, BTA3, BTA5, BTA6, BTA7, BTA10, BTA11, BTA13, BTA14, BTA15, BTA16, BTA19, BTA20, BTA21, BTA24, BTA28), explaining a minimum additive genetic variance of 0.135% at BTA21 and maximum additive genetic variance of 0.237% at BTA20. Together, these windows explained 3.087% of the total additive genetic variance for BW. The top 1% windows, along with their genomic positions, overlapped 286 positional candidate genes, classified as protein-coding (n = 212), long noncoding RNAs (n = 49), small noncoding RNAs (n = 5), miscellaneous noncoding RNAs (n = 1), micro-RNA (n = 5), small nuclear RNA (n = 9), and pseudogene (n = 5) as detailed in the Supplementary Table S2A. In summary, a total of 16 biological processes, 10 cellular components, one molecular function; and one KEGG pathway are statistically associated with the genes related to birthweight in Nellore and highlighted in the Supplementary Table S2B.

#### 3.1.3 Body conformation

The top 1% genomic window were located across 17 chromosomes as seen in Figure 1c (i.e., BTA2, BTA3, BTA5, BTA6, BTA7, BTA8, BTA10, BTA11, BTA12, BTA14, BTA16, BTA19, BTA20, BTA21, BTA22, BTA26), explaining a minimum additive genetic variance of 0.129% at BTA20 and maximum additive genetic variance of 0.208% at BTA5. Together, these windows explained 2.964% of the total additive genetic variance for BC. The top 1% windows, along with their genomic positions, overlapped 991 positional candidate genes, classified as protein-coding (n = 637), long noncoding RNAs (n = 263), small noncoding RNAs (n = 14), miscellaneous noncoding RNAs (n = 2), micro-RNA (n = 17), small nuclear RNA (n = 45), and pseudogene (n = 11) as detailed in the Supplementary Table S3A. In summary, a total of five biological processes, and two molecular functions are statistically associated with the genes related to body conformation in Nellore as highlighted in the Supplementary Table S3B.

#### 3.1.4 Expected progeny difference for weight at 120 days

The top 1% genomic window were located across 14 chromosomes as seen in Figure 1d (i.e., BTA2, BTA3, BTA6, BTA7, BTA9, BTA11, BTA13, BTA14, BTA17, BTA22, BTA25, BTA26, BT28, BTA29), explaining a minimum additive genetic variance of 0.134% at BTA29 and maximum additive genetic variance of 0.176% at BTA14. Together, these windows explained 2.978% of the total additive genetic variance for EPD_120. The top 1% windows, along with their genomic positions, overlapped 239 positional candidate genes, classified as protein-coding (N = 158), long noncoding RNAs (n = 50), small noncoding RNAs (n = 9), micro-RNA (n = 1), small nuclear RNA (n = 16), and pseudogene (n = 4) as detailed in the Supplementary Table S4A. In summary, a total of two biological process, two cellular process and four molecular functions were statistically associated with the genes related to EPD120 in Nellore as highlighted in the Supplementary Table S4B.

#### 3.1.5 Finishing score

The top 1% genomic window were located across 14 chromosomes as seen in Figure 1e (i.e., BTA2, BTA3, BTA4, BTA6, BTA8, BTA9, BTA10, BTA11, BTA13, BTA14, BTA20, BTA21, BTA23), explaining a minimum additive genetic variance of 0.129% at BTA11 and maximum additive genetic variance of 0.240% at BTA14. Together, these windows explained 3.008% of the total additive genetic variance for FS. The top 1% windows, along with their genomic positions, overlapped 254 positional candidate genes, classified as protein-coding (n = 177), long noncoding RNAs (n = 51), micro-RNA (n = 7), small nuclear RNA (n = 12), and pseudogene (n = 5) as detailed in the Supplementary Table S5A. In summary, a total of six biological process, two cellular process; and two molecular functions were statistically associated with the genes related to finishing score in Nellore as highlighted in the Supplementary Table S5B.

#### 3.1.6 Marbling

The top 1% genomic window were located across 13 chromosomes as seen in Figure 1f (i.e., BTA1, BTA2, BTA5, BTA6, BTA7, BTA8, BTA9, BTA12, BTA14, BTA17, BTA22, BTA23, BTA27), explaining a minimum additive genetic variance of 0.128% at BTA12 and maximum additive genetic variance of 0.253% at BTA6. Together, these windows explained 2.995% of the total additive genetic variance for MARB. The top 1% windows, along with their genomic positions, overlapped with 368 positional candidate genes, classified as protein-coding (n = 281), long noncoding RNAs (n = 53), micro-RNA (n = 12), small nuclear RNA (n = 10), small noncoding RNA (n = 8), and pseudogene (n = 3) as detailed in the Supplementary Table S6A. In summary, a total of three biological process and two molecular functions were statistically associated with the genes related to marbling in Nellore as highlighted in the Supplementary Table S6B.

#### 3.1.7 Muscularity

The top 1% genomic windows were located across 15 chromosomes as seen in Figure 1g (i.e., BTA3, BTA5, BTA6, BTA8, BTA9, BTA10, BTA11, BTA13, BTA14, BTA15, BTA16, BTA20, BTA23, BTA27, BTA28), explaining a minimum additive genetic variance of 0.126% at BTA3 and maximum additive genetic variance of 0.232% at BTA6. Together, these windows explained 3.013% of the total additive genetic variance for MUSC. The top 1% windows, along with their genomic positions, overlapped with 202 positional candidate genes, classified as protein-coding (n = 129), long noncoding RNAs (n = 54), micro-RNA (n = 3), small nuclear RNA (n = 11), and pseudogene (n = 2) as detailed in the Supplementary Table S7A. In summary, only one biological process, and one molecular function were statistically associated with the genes related to muscularity in Nellore as highlighted in the Supplementary Table S7B.

#### 3.1.8 Ribeye area

The top 1% genomic window were located across 13 chromosomes as seen in Figure 1h (i.e., BTA1, BTA3, BTA4, BTA6, BTA8, BTA9, BTA10, BTA11, BTA14, BTA15, BTA20, BTA21, BTA23), explaining a minimum additive genetic variance of 0.125% at BTA10 and maximum additive genetic variance of 0.186% at BTA1. Together, these windows explained 2.962% of the total additive genetic variance for REA. The top 1% windows, along with their genomic positions, overlapped with 238 positional candidate genes, classified as protein-coding (n = 157), long noncoding RNAs (n = 52), small noncoding RNAs (n = 8), micro-RNA (n = 6), small nuclear RNA (n = 12), and pseudogene (n = 3) as detailed in the Supplementary Table S8A. In summary, one molecular function, and one KEGG pathway were statistically associated with the genes related to ribeye area in Nellore as highlighted in the Supplementary Table S8B.

#### 3.1.9 Finishing precocity

The top 1% genomic window were located across 13 chromosomes as seen in Figure 1i (i.e., BTA1, BTA3, BTA6, BTA7, BTA8, BTA9, BTA10, BTA14, BTA16, BTA20, BTA21, BTA23, BTA27), explaining a minimum additive genetic variance of 0.130% and maximum additive genetic variance of 0.243% at BTA6. Together, these windows explained 3.158% of the total additive genetic variance for FP. The top 1% windows, along with their genomic positions, overlapped with 217 positional candidate genes, classified as protein-coding (n = 136), long noncoding RNAs (n = 60), small noncoding RNAs (n = 3), miscellaneous noncoding RNAs (n = 1), micro-RNA (n = 6), and small nuclear RNA (n = 12) as detailed in the Supplementary Table S9A. In summary, one biological process, and one KEGG pathway, were statistically associated with the genes related to finishing precocity in Nellore as highlighted in the Supplementary Table S9B.

#### 3.1.10 Scrotal circumference at 365 days

The top 1% genomic window were located across 14 chromosomes as seen in Figure 1j (i.e., BTA1, BTA2, BTA3, BTA5, BTA6, BTA7, BTA8, BTA9, BTA10, BTA20, BTA21, BTA23, BTA27, BTA28), explaining a minimum additive genetic variance of 0.130% at BTA27 and maximum additive genetic variance of 0.197% at BTA6. Together, these windows explained 3.005% of the total additive genetic variance for SC365. The top 1% windows, along with their genomic positions, overlapped with 286 positional candidate genes, classified as protein-coding (n = 192), long noncoding RNAs (n = 52), small noncoding RNAs (n = 7), micro-RNA (n = 3), small nuclear RNA (n = 9), and pseudogene (n = 4) as detailed in the Supplementary Table S10A. In summary, a total of four biological process, five molecular functions, and one KEGG pathway, were statistically associated with the genes related to scrotal circumference at 365 days in Nellore as highlighted in the Supplementary Table S10B.

#### 3.1.11 Scrotal circumference at 450 days

The top 1% genomic window were located across 15 chromosomes as seen in Figure 1k (i.e., BTA1, BTA5, BTA6, BTA7, BTA8, BTA9, BTA10, BTA15, BTA21, BTA23, BTA25, BTA27, BTA28), explaining a minimum additive genetic variance of 0.130% at BTA8 and maximum additive genetic variance of 0.197% at BTA10. Together, these windows explained 3.005% of the total additive genetic variance for SC450. The top 1% windows, along with their genomic positions, overlapped with 271 positional candidate genes, classified as protein-coding (n = 184), long noncoding RNAs (n = 68), small noncoding RNAs (n = 4), micro-RNA (n = 2), small nuclear RNA (n = 7), and pseudogene (6) as detailed in the Supplementary Table S11A. In summary, a total of seven biological processes, five molecular functions, two cellular components, and one KEGG pathway, were statistically associated with the genes related to scrotal circumference at 450 days in Nellore as highlighted in the Supplementary Table S11B.

#### 3.1.12 Stayability

The top 1% genomic windows were located across 12 chromosomes as seen in Figure 1l (i.e., BTA4, BTA5, BTA6, BTA9, BTA14, BTA15, BTA16, BTA17, BTA18, BTA23, BTA29), explaining a minimum additive genetic variance of 0.123% and maximum additive genetic variance of 0.164% at BTA5. Together, these windows explained 2.779% of the total additive genetic variance for STAY. The top 1% windows, along with their genomic positions, overlapped with 215 positional candidate genes, classified as protein-coding (n = 143), long noncoding RNAs (n = 32), small noncoding RNAs (n = 2), micro-RNA (n = 8), small nuclear RNA (n = 26), and pseudogene (n = 4) as detailed in the Supplementary Table S12A. In summary, a total of five biological process, four molecular functions, two cellular component, and three KEGG pathways, were statistically associated with the genes related to stayability in Nellore as highlighted in the Supplementary Table S12B.

#### 3.1.13 Weight at 120 days of age

The top 1% genomic window were located across 12 chromosomes as seen in Figure 1m (i.e., BTA3, BTA5, BTA6, BTA7, BTA8, BTA10, BTA11, BTA14, BTA16, BTA19, BTA20, BTA28), explaining a minimum additive genetic variance of 0.125% at BTA8 and maximum additive genetic variance of 0.218% at BTA10. Together, these windows explained 2.995% of the total additive genetic variance of W120. The top 1% windows, along with their genomic positions, overlapped with 373 positional candidate genes, classified as protein-coding (n = 273), long noncoding RNAs (n = 58), small noncoding RNAs (n = 6), miscellaneous noncoding RNAs (n = 1), micro-RNA (n = 10), small nuclear RNA (n = 16), and pseudogene (n = 8) as detailed in the Supplementary Table S13A. In summary, a total of nine biological process, four cellular components, three molecular functions, and one KEGG pathway, were statistically associated with the genes related to weight at 120 days in Nellore as highlighted in the Supplementary Table S13B.

#### 3.1.14 Weight at 210 days of age

The top 1% genomic windows were located across 14 chromosomes as seen in Figure 1n (i.e., BTA3, BTA5, BTA6, BTA7, BTA8, BTA10, BTA11, BTA14, BTA16, BTA19, BTA20, BTA21, BTA23, BTA28), explaining a minimum additive genetic variance of 0.127% at BTA28 and maximum additive genetic variance of 0.212% at BTA10. Together, these windows explained 2.980% of the total additive genetic variance of W210. The top 1% windows, along with their genomic positions, overlapped with 305 positional candidate genes, classified as protein-coding (n = 221), long noncoding RNAs (n = 54), small noncoding RNAs (n = 4), micro-RNA (n = 4), small nuclear RNA (n = 15), and pseudogene (n = 6) as detailed in the Supplementary Table S14A. In summary, a total of 10 biological process, five cellular component, four molecular functions, and two KEGG pathways, were statistically associated with the genes related to weight at 210 days in Nellore as highlighted in the Supplementary Table S14B.

#### 3.1.15 Weight at 365 days of age

The top 1% genomic window were located across 14 chromosomes as seen in Figure 1o (i.e., BTA3, BTA5, BTA6, BTA8, BTA10, BTA11, BTA14, BTA16, BTA19, BTA20, BTA21, BTA23, BTA27, BTA28), explaining a minimum additive genetic variance of 0.124% at BTA8 and maximum additive genetic variance of 0.190%. At BTA6. Together, these windows explained 2.869% of the total additive genetic variance of W365. The top 1% windows, along with their genomic positions, overlapped with 351 positional candidate genes, classified as protein-coding (n = 244), long noncoding RNAs (n = 64), small noncoding RNAs (n = 4), micro-RNA (n = 3), small nuclear RNA (n = 32), and pseudogene (n = 3) as detailed in the Supplementary Table S15A. In summary, a total of 10 biological process, eight cellular component, nine molecular functions, and three KEGG pathways, were statistically associated with the genes related to weight at 365 days in Nellore as highlighted in the Supplementary Table S15B.

#### 3.1.16 Weight at 450 days of age

The top 1% genomic windows were located across 17 chromosomes as seen in Figure 1p (i.e., BTA1, BTA2, BTA3, BTA5, BTA6, BTA7, BTA10, BTA11, BTA13, BTA14, BTA15, BTA16, BTA19, BTA20, BTA21, BTA24, BTA28), explaining a minimum additive genetic variance of 0.125% at BTA21and maximum additive genetic variance of 0.196% at BTA6. Together, these windows explained 2.867% of the total additive genetic variance of W450. The top 1% windows, along with their genomic positions, overlapped with 352 positional candidate genes, classified as protein-coding (n = 245), long noncoding RNAs (n = 58), small noncoding RNAs (n = 4), micro-RNA (n = 5), small nuclear RNA (n = 35), and pseudogene (n = 4) as detailed in the Supplementary Table S15C. In summary, a total of 13 biological process, seven cellular component, eight molecular functions, and three KEGG pathways, were statistically associated with the genes related to weight at 450 days in Nellore as highlighted in the Supplementary Table S15D.

## 4 Discussion

### 4.1 Use of APY for GWAS

Using a large number of genotyped animals in GWAS to identify candidate genes and genomic regions associated with key traits can increase the power of the study and lead to a better understanding of the genetic architecture underlying these traits (Li et al., 2021). While several studies have investigated genomic regions associated with important traits in Nellore cattle, none of these studies have used datasets with over 65,000 genotyped Nellore animals (Schmidt et al., 2023). Our study, however, leverages the results from a single-step GEBV approach with over 300,000 genotyped animals and more than 14 million pedigree records to identify the candidate genes associated with key traits in Nellore cattle.

A major challenge when using large numbers of genotyped animals in GWAS is the computational infeasibility of directly inverting **G** for large populations. This limitation necessitates the use of APY, which provides a computationally efficient method for creating and storing the inverse of **G**. While few studies have applied APY in GWAS, they have focused on the Holstein and Angus breeds (e.g., Leite et al., 2024; Aguilar et al., 2019; Misztal et al., 2024), and none have used that for Nellore.

### 4.2 Identification of candidate genes for each trait

This section presents a detailed discussion of the key candidate genes identified within each trait, emphasizing their potential roles in the genetic architecture of the analyzed phenotypes in Nellore cattle. By focusing on the top 1% of genomic windows, we highlighted regions with the highest additive genetic variance contributions, while selectively discussing a subset of candidate genes from the broader list of candidate genes that are likely influencing the polygenicity of these specific traits.

#### 4.2.1 Age at first calving

Age at first calving is a key reproductive trait incorporated into beef cattle breeding programs as an indicator of sexual precocity in female cattle (Schmidt et al., 2018; Alves et al., 2022). Age at first calving is calculated as the difference in months between the dam’s date of birth and the date of birth of her first registered calf (Eler et al., 2014). Sires with favorable breeding values for AFC are preferred in selection, as this contributes to a shorter generation interval, thereby enabling earlier calving in their progeny (Eler et al., 2014). The top 1% genomic windows were spread in 15 chromosomes collectively explaining 3.012% of the additive genetic variance for this trait with the regions responsible for coding 225 genes as seen in Table 1. These results corroborate with the findings reported by Sbardella et al. (2021), who identified genomic windows (containing 10 consecutive SNPs, population genotyped using 50 K) explaining 0.20%–1.11% of the additive genetic variance for AFC (Sbardella et al., 2021).

**TABLE 1:** Candidate genes located within the top 1% genomic windows associated with age at first calving in Nellore cattle.

BTA	Position (BP)	Candidate genes	^1^Var (%)
6	69,258,860–70,084,303	*CHIC2, GSX2, PDGFRA, U6, bta-mir-4449*	0.235
10	85,348,496–86,364,230	*COQ6, ENTPD5, BBOF1, ALDH6A1, LIN52, VSX2, ABCD4, VRTN, SYNDIG1L, NPC2, ISCA2, LTBP2, AREL1. FCF1, YLPM1, PROX2, DLST, RPS6KL1, PGF, EIF2B2, ACYP1, ZC2HC1C, NEK9, Metazoa_SRP, MLH3*	0.193
21	58,658,243–59,478,510	*OTUB2, DDX24, ISG12(B), IFI27, IFI27L2, PPP4R4, SERPINA10, SERPINA6, SERPINA1, SERPINA11, SERPINA14, SERPINA12, SERPINA5, SERPINA3-3*	0.185
9	80,031,480–80,701,610	*HIVEP2, AIG1, bta-mir-2284aa-4*	0.171
14	41,537,492–42,576,327	*PKIA, IL7*	0.158
16	58,877,604–59,420,135	*SEC16B*	0.147
8	57,399,938–58,531,522	*TLE1*	0.146
21	35,060,169–35,613,940	*STXBP6*	0.146
4	90,187,337–90,743,037	*GRM8*	0.142
14	10,578,647–11,171,344	*GSDMC, CYRIB, ASAP1*	0.140
27	9,374,334–10,343,439	*-*	0.140
7	54,999,335–56,005,879	*YIPF5, KCTD16*	0.137
10	82,912,907–83,689,406	*SIPA1L1, RGS6*	0.137
20	9,527,068–10,621,897	*BDP1, SERF1A, GTF2H2, TAF9, OCLN, MARVELD2, RAD17, AK6, CCDC125, CDK7, CENPH, CCNB1, SLC30A5, bta-mir-7858, bta-mir-11986b, SMN2, NAIP, CARTPT, MCCC2*	0.136
6	67,663,811–68,546,478	*CWH43, DCUN1D4* *LRRC66, SGCB, SPATA18, USP46, RASL11B, SCFD2*	0.135
28	8,749,119–9,703,912	*EDARADD, LGALS8, HEATR1, ACTN2, MTR, NID1, RYR2, GPR137B, ERO1B, U6*	0.135
4	84,699,677–85,581,389	*KCND2, CPED1, TSPAN12, ING3*	0.135
5	9,286,252–10,492,733	*MYF6, MYF5, LIN7A, PPP1R12A, OTOGL, PTPRQ*	0.134
11	90,288,247–91,116,303	*SOX11*	0.132
2	68,983,135–71,193,227	*EN1, MARCO, C1QL2, STEAP3, C2H2orf76, DBI, INSIG2, DDX18, CCDC93*	0.130

Proportion of the trait variance explained by each window (%).

A study by Dubon et al. (2021) investigating novel candidate genes for AFC in Nellore cows, identified a genomic region between 58.9–59.7 Mb on BTA21, which closely aligns with the 58.6–59.4 Mb genomic region identified in our study (Dubon et al., 2021). The latter author suggested this area as a potential novel region influencing AFC, and both studies found that this region encodes *SERPINs*, a superfamily of protease-inhibiting proteins (Heit et al., 2013). Notably, *SERPINA14*, a hormonally induced protein secreted by endometrial epithelium during pregnancy, was highlighted as a potential candidate gene in this region (Padua et al., 2010). The role of *SERPINA14* in pregnancy, particularly in relation to AFC, was further evidenced by Loux et al. (2019), who observed higher expression of *SERPINA14* in the cervical muscosa and endometrium of mares during pregnancy than in estrus (Loux et al., 2019). In addition, *SERPINA5*, another gene in this region, showed higher expression in healthy follicles compared to atretic follicles, which are crucial for reproduction (Hayashi et al., 2011). Another relevant gene, *SERPINA1*, is associated with accelerated follicular growth, as cows with 30% lower *SERPINA1* levels in follicular fluids exhibited reduced fertility compared to cows with normal fertility (Zachut et al., 2016).

A significant gene identified between the 69.2 and 70.0 Mb genomic region of BTA 6 is the *NEK9* gene, a NIMA family protein kinase activated in mitosis by the binding of *NEK6* and *NEK7* (Belham et al., 2003). A likely novel candidate gene was also found in this genomic window: *CCNB1*, which is essential for spindle checkpoint regulation, meiosis, and mitosis (Tang et al., 2017; Alfonso-Pérez et al., 2019). Another novel candidate gene *CARTPT* highlighted in 9.5–10.6 Mb on BTA20 is seen to be expressed in the oocyte and granulosa cell of the follicle (Juengel et al., 2017). Additional candidate genes identified in this analysis are related to functions such as molecular function regulator (GO:0098772), serine-type endopeptidase inhibitor (GO:0004867), peptidase regulator (GO:0061134), and enzyme regulator (GO:0030234). These genomic regions enhance the phenotypic expression of AFC by containing genes that regulate critical processes like meiosis and follicular development, improving reproductive efficiency and enabling earlier first calving in cattle.

#### 4.2.2 Birth weight

Birth weight is an economically important trait in beef cattle, as it is usually the first trait recorded in calves (Utsunomiya et al., 2013) and is closely related to growth traits (Boligon et al., 2009). It is also an important selection criterion for enhancing calving ease and adult body weight (Bourdon and Brinks, 1982; Eriksson et al., 2004). The top 1% genomic windows were found in 17 collectively explaining 3.087% of the additive genetic variance for birth weight and responsible for coding 286 genes as seen in Table 2. Carvalho et al. (2020a) and Terakado et al. (2018) reported genomic windows explaining 2.32% and ∼4%, respectively, for BW (Terakado et al., 2018; Carvalho et al., 2020a).

**TABLE 2:** Candidate genes located within the top 1% genomic windows associated with birth weight in Nellore cattle.

BTA	Position (BP)	Candidate	^1^Var (%)
20	46,600,025–47,861,825	*CDH9*	0.237
19	40,595,675–42,117,748	*TOP2A, IGFBP4, TNS4, KRT222, CCR7, KRT27, KRT24, KRT10, JUP, P3H4, FKBP10, NT5C3B, KLHL10, ACLY, ODAD4, KRT12, KRT20, KRT23, KRT39, KRT40, HAP1, GAST, KRT14, KRT9, KRT16, GJD3, EIF1, KRTAP4-7, KRTAP9-2, KRTAP16-1, KRT31, KRT37, KRT36, KRTAP3-3, KRTAP3-1, KRTAP17-1, U6, bta-mir-2285cb, SMARCE1, KRT28, KRT25, KLHL11, KRT17, KRT42, KRT19, KRT15, KRT33A, KRT32*	0.170
6	69,176,844–69,912,628	*CHIC2, GSX2, PDGFRA, U6*	0.169
5	39,344,450–40,374,767	*PDZRN4, CNTN1*	0.169
10	82,906,890–83,684,844	*SIPA1L1, RGS6, Metazoa_SRP*	0.160
13	24,138,006–25,149,812	*MSRB2, PTF1A, OTUD1, KIAA1217*	0.160
3	107,633,453–108,718,037	*SF3A3, INPP5B, MTF1, C3H1orf122, YRDC, MANEAL, POU3F1, UTP11, FHL3, CDCA8, AIRIM, EPHA10, ZC3H12A, RSPO1, SNIP1, GNL2, DNALI1, U6, MEAF6*	0.160
1	62,577,819–63,381,533	*-*	0.159
2	130,353,122–131,738,344	*WNT4, CDC42, CELA3B, HSPG2, USP48, RAP1GAP, ALPL, ECE1, EIF4G3, U6, U6*	0.152
14	41,193,791–42,165,679	*PKIA, IL7, U6*	0.148
20	52,696,774–53,820,596	*CDH18, bta-mir-2361*	0.148
16	76,515,256–77,345,088	*DENND1B, C16H1orf53, LHX9, NEK7*	0.145
24	51,290,309–52,129,809	*DCC*	0.144
7	66,385,051–67,215,595	*U6*	0.143
15	70,947,433–71,871,075	*-*	0.139
21	49,649,455–50,547,021	*-*	0.139
11	73,959,275–75,270,350	*DNMT3A, POMC, EFR3B, DNAJC27, ADCY3, CENPO, PTRHD1, NCOA1, ITSN2, FAM228A, FAM228B, PFN4, TP53I3, SF3B6, FKBP1B, WDCP, MFSD2B, UBXN2A, ATAD2B, KLHL29, U3, bta-mir-1301*	0.139
28	43,804,023–45,132,821	*MARCHF8, ALOX5, ZNF22, DEPP1, RASSF4, TMEM72, CXCL12, CHAT, C28H10orf53, OGDHL, PARG, TIMM23, NCOA4, MSMB, FAM21A, ZFAND4, OR13A1, OR6D16, SNORA74*	0.136
14	9,011,203–9,987,316	*KCNQ3, HHLA1, OC90, EFR3A, ADCY8*	0.136
21	59,196,055–59,997,988	*SERPINA12, SERPINA5, DICER1, bta-mir-2365, U6, bta-mir-2284a, SERPINA3-3*	0.135

Proportion of the trait variance explained by each window (%).

A study by Carvalho et al. (2020b) performed to identify regions associated with BW in Nellore cattle recognized important genomic regions: 49.0–50.0 Mb on BTA14 and 52.1–52.6 Mb on BTA21, which closely aligns with 41.19–42.17 Mb on BTA14 and 49.6–50.5 Mb on BTA 21 identified in our study (Carvalho et al., 2020b). Different regions of BTA14 were associated with stature in humans by Utsunomiya et al. (2013) and birthweight in Nellore cattle by Terakado et al. (2018) (Utsunomiya et al., 2013; Terakado et al., 2018), however in our study, the genomic region 41.19–42.17 Mb on BTA14 was seen to highlight *IL7* (Interleukin-7), a gene known to be associated with cell differentiation and cellular development, particularly in B and T cell maturation (Chen et al., 2021).

The candidate gene *SF3A3,* identified in our study and located in the 107.6–108.7 Mb region of BTA3, encodes a splicing factor 3a subunit 3, which has been associated with body weight in Indonesia cattle breed (Putra et al., 2024).

Another notable candidate gene *HSPG2* (Perlecan), spanning 130.4–131.7 Mb of BTA2, is responsible for the anatomical structure morphogenesis, and reinforces its role in tissue morphogenesis, differentiation. And development as this process plays a unique role in organ function thereby influencing growth and weight of animal at birth (Ocken et al., 2020). Additional candidate genes highlighted in this study for BW are related to cellular developmental process (GO:0048869), cell differentiation (GO:0030154), tissue development (GO:0009888), epithelium development (GO:0060429), cytoskeleton organization (GO:0007010), epithelial cell differentiation (GO:0030855), skin development (GO:0043588), and cellular component organization (GO:0016043). These genomic regions contribute to the phenotypic variation in birth weight by containing genes that regulate key biological processes such as cellular differentiation, and tissue morphogenesis, which influences development and overall body size in cattle.

#### 4.2.3 Body conformation

Body conformation traits are widely used as selection criteria in Zebu cattle breeding programs due to their strong association with carcass quality (Shiotsuki et al., 2009; Machado et al., 2022). Body conformation is also closely related to health, productivity, and cattle longevity (Wu et al., 2013; Machado et al., 2022). The top 1% genomic windows for body conformation were spread in 17 chromosomes collectively explaining 2.964% of the additive genetic variance for this trait. Similarly, Carreño et al. (2019) and Machado et al. (2022) reported that the highest proportion of additive genetic variance explained by the genomic windows was less than 0.5% and 4%, respectively, for body conformation (Carreño et al., 2019; Machado et al., 2022). The top 1% of genomic windows in our study were responsible for coding 991 genes as seen in Table 3.

**TABLE 3:** Candidate genes located within the top 1% genomic windows associated with body conformation in Nellore cattle.

BTA	Position	Candidate genes	^1^Var (%)
5	39,344,450–40,374,767	*PDZRN4, CNTN1*	0.208
16	60,596,486–61,534,166	*SOAT1, AXDND1, NPHS2, TDRD5, FAM163A, TOR1AIP2, TOR1AIP1, CEP350, QSOX1, LHX4, ACBD6, U6, U2*	0.207
10	82,899,590–83,683,111	*SIPA1L1, RGS6, Metazoa_SRP*	0.184
8	80,786,825–81,426,962	*DAPK1, CTSL, FBP2, AOPEP, FBP1*	0.177
7	66,097,903–66,885,302	*GEMIN5, MRPL22, U6, U2, SNORA70, U6*	0.156
11	70,009,223–70,492,069	*FAM161A, REG3A, CCT4, B3GNT2, COMMD1, REG3G, SUCLG1, CTNNA2, LRRTM1, LRRTM4, C11H2orf74, AHSA2, USP34, XPO1, U1, SNORA72, U6, U6, U1, Metazoa_SRP, 5S_rRNA, SNORA72, LGALSL, AFTPH, ACTR2, SPRED2, GFPT1, C1D, PPP3R1, PNO1, CNRIP1, NFU1, AAK1, SERTAD2, PLEK, TMEM17, EHBP1, PCYOX1, SNRPG, EHD3, CAPN14, SIX3, GALNT14, RMDN2, FBXO48, APLF, OTX1, WDPCP, MDH1, CYP1B1, LRPPRC, UGP2, VPS54, PELI1, ANXA4, GMCL1, SIX2, PLEKHH2, MXD1, PCBP1, C11H2orf42, DYNC2LI1, TIA1, ABCG5, PROKR1, ABCG8, ARHGAP25, EPAS1, BMP10, GKN2, GKN1, SRBD1, ANTXR1, CAPN13, PRKCE, LCLAT1, EPCAM, MEIS1, SLC10A6, PPM1B, LBH, MSH2, YPEL5, KCNK12, MSH6, ALK, FBXO11, SLC1A4, TMEM247, ETAA1, ATP6V1E2, RHOQ, CEP68, PIGF, RAB1A, CRIPT, SOCS5, MCFD2, TTC7A, ASPRV1, STPG4, CALM2, OXER1, HAAO, ZFP36L2, THADA, DNAAF10, ATL2, SLC3A1, PREPL, CAMKMT, ARHGEF33, SOS1, CDKL4, MAP4K3, TMEM178A, THUMPD2, SLC8A1, HNRNPLL, GALM, SRSF7, DHX57, MORN2, PKDCC, EML4, COX7A2L, KCNG3, MTA3, VIT, HEATR5B, GPATCH11, EIF2AK2, SULT6B1, CEBPZOS, CEBPZ, NDUFAF7, PRKD3, QPCT, FAM136A, XDH, IL1RL2, IL1RL1, IL18R1, IL18RAP, SLC9A4, CDC42EP3, SLC9A2, MFSD9, TMEM182, CRIM1, FEZ2, SRD5A2, MEMO1, MRPS9, FAM98A, TGFBRAP1, DPY30, SPAST, SPR, DYSF, ZNF638, PAIP2B, NAGK, TEX261, ANKRD53, ATP6V1B1, VAX2, SLC4A5, MTHFD2, MOB1A, EXOC6B, CD207, BOLA3, CLEC4F, FIGLA, C11H2orf49, ADD2, FHL2, SLC30A6, TACR1, NLRC4, YIPF4, TTC27, TET3, DGUOK, ACTG2, STAMBP, C11H2orf78, DUSP11, TPRKB, ALMS1, TGFA, LTBP1, RASGRP3, EGR4, FBXO41, CYP26B1, CCT7, PRADC1, SMYD5, NOTO, RAB11FIP5, SFXN5, EMX1, DOK1, LOXL3, HTRA2, AUP1, DQX1, TLX2, PCGF1, LBX2, POLE4, HK2, SEMA4F, M1AP, POU3F3, CCDC142, MRPL53, MOGS, WBP1, RTKN, WDR54, C11H2orf81, MGC152281, DCTN1, STRN, BIRC6, INO80B, ACYP2, C11H2orf73, SPTBN1, EML6, RTN4, FOXN2, STON1, GTF2A1L, LHCGR, FSHR, RPS27A, MTIF2, PRORSD1, CCDC88A, NRXN1, CFAP36, PPP4R3B, PNPT1, EFEMP1, CCDC85A, ASB3, CHAC2, ERLEC1, GPR75, PSME4, PPP1R21, CLHC1, EDAR, VRK2, FANCL, PSD4, SLC5A7, CCDC138, RANBP2, SH2D6, CAPG, BCL11A, PAPOLG, REL, PUS10, PEX13, SANBR, ATOH8, RNF103, CHMP3, ELMOD3, RETSAT, TCF7L1, GCFC2, MRPL19, LIMS1, PAX8, KCMF1, RMND5A, ST6GAL2, TMSB10, DNAH6, KDM3A* *GCC2, EVA1A, SEPTIN10, SH3RF3, ECRG4, REEP1, MRPL35, IMMT, SULT1C2, SULT1C3, UXS1, EIF2AK3, SPMIP9, FOXI3, PTCD3, POLR1A, RPIA, THNSL2, ST3GAL5, SFTPB, NCK2, TTL, POLR1B, USP39, C11H2orf68, RNF181, TMEM150A, VAMP8, GGCX, MAT2A, CHCHD5, SLC20A1, IL1B, IL37, IL36G, IL36A, IL36RN, IL1F10, IL1RN, FABP1, SMYD1, KRCC1, CD8B, CD8A, SOWAHC, SNORD94, SULT1C4, VAMP5*	0.149
19	55,766,113–56,804,981	*GRB2, SLC25A19, MIF4GD, MRPS7, GGA3, NUP85, SUMO2, NT5C, ARMC7, SLC16A5, KCTD2, ATP5PD, HID1, OTOP3, OTOP2, USH1G, FADS6, FDXR, MRPL58, CDR2L, WBP2, UNC13D, UNK, H3-3B, SAP30BP, RECQL5, SMIM6, SMIM5, GRIN2C, TMEM104, NAT9, NHERF1, RAB37, CD300E, LLGL2, TSEN54, CASKIN2, GALK1, ITGB4, JPT1, TMEM94*	0.146
3	1,728,119–2,684,730	*GPA33, MAEL, ILDR2, TADA1, POGK*	0.142
3	95,973,132–97,127,451	*ELAVL4*	0.138
21	49,673,221–50,607,534	*-*	0.136
3	32,225,556–33,265,065	*CD53, KCNA3, LAMTOR5, KCNA2, KCNA10, CYM, SLC16A4, RBM15, PROK1, CEPT1, DRAM2, LRIF1, KCNC4, SLC6A17*	0.135
26	42,777,934–43,470,582	*IKZF5, PSTK, ACADSB, HMX3, HMX2, BUB3, GPR26*	0.135
2	120,468,635–121,510,489	*SYNC, RBBP4, ZBTB8OS, ZBTB8A, ZBTB8B, BSDC1, TSSK3, FAM229A, MARCKSL1, HDAC1, LCK, FAM167B, MTMR9, EIF3I, TMEM234, DCDC2B, IQCC, CCDC28B, CLDN12, SHOX, TRIM62, AZIN2, AK2, RNF19B, TMEM54, HPCA, FNDC5, S100PBP, YARS1, KIAA1522*	0.135
6	69,176,844–69,912,628	*CHIC2, GSX2, PDGFRA*	0.133
12	84,446,733–85,726,570	*COL4A1, COL4A2, RAB20, NAXD, CARS2, ING1, IRS2, ANKRD10, ARHGEF7, TEX29*	0.133
14	34,928,777–36,067,306	*EYA1, MSC, TRPA1, KCNB2*	0.132
22	31,136,273–32,182,398	*MDFIC2, MITF*	0.131
14	10,584,341–11,176,477	*GSDMC, CYRIB, ASAP1*	0.130
5	40,610,629–41,720,384	*SLC2A13, C12orf40, ABCD2, LRRK2*	0.129
20	30,540,195–31,684,103	*NIM1K, ZNF131, FGF10, NNT, PAIP1, C20H5orf34, TMEM267, CCL28, HMGCS1*	0.129

Proportion of the trait variance explained by each window (%).

In both our study and that by Machado et al. (2022), the genomic region on BTA14 between 34.9 and 36.1 Mb highlighted the *KCNB2* gene, a member of the potassium channel family primarily expressed in smooth muscle cells and critical for potassium ion transport across cell membranes (Bhat et al., 2024). The candidate gene *ILDR2* identified in the genomic window 1.72–2.68 Mb of BTA 3, is a member of the B7 family of immunomodulatory receptors. This gene is expressed in immune cells and plays an important role in cellular metabolic and biosynthetic processes, including homeostasis (Watanabe et al., 2013). Another notable candidate gene on BTA3 is *SLC6A17*, found in the 32.22–33.27 Mb window of BTA 3, which is a member of the *SLC6* family. The *SLC6* family is known to act as transporters for neurotransmitters and amino acid, playing an important role in the regulation of glutamatergic synapses (Iqbal et al., 2015). The likely novel candidate gene, *CNTN1*, annotated in the genomic window 39.34–40.37 Mb of BTA 5, is from the *CTNTN* family and mediates cell surface interactions essential for nervous system development which can indirectly affect structural organization, thereby resulting in notable changes in body conformation (Um and Ko, 2017).

In the genomic window 70.09–70.49 Mb of BTA 11, three candidate genes, *WDPCP*, *SFTPB*, and *SERTAD* were identified. The *WDPCP* gene is involved in ciliogenesis, a process of building cilia necessary for cell signaling, regulation of cellular proliferation, and differentiation of developing limbs (Langhans et al., 2021). Loss of *WDPCP* function has been linked to developmental defects in the heart, neural tube, and limbs (Langhans et al., 2021). The *SFTPB* gene encodes surfactant protein B (SP-B), a protein known to help with lung function and homeostasis (Leibel et al., 2019). Interestingly, *SFTPB* deficiency in newborns can lead to lethal respiratory distress syndrome (Leibel et al., 2019). Another candidate gene in this region, *SERTAD2*, is a member of the *SERTAD* family, known to be involved in the regulation of cell growth. This gene has also been found to be prioritized as a functional gene for reproduction traits in our previous study (Ogunbawo et al., 2024). This finding highlights the gene’s pleiotropic effect, which may influence the expression of both body conformation and reproductive traits (Darwish et al., 2007; Ogunbawo et al., 2024).

Gene Ontology terms related to body conformation include ear morphogenesis (GO:0042471) and inner ear morphogenesis (GO:0042472), processes that generate and organize the ear’s anatomical structures, and may contribute to crucial components of body conformation (de Haan et al., 2024; Li et al., 2023). The *PAX eight* gene, associated GO terms (GO:0044237, GO:0008152) play a fundamental role in cell differentiation (Di Palma et al., 2013). Additional candidate genes highlighted in this study for BC are related to post-Golgi vesicle-mediated transport (GO:0006892) and Golgi to plasma membrane transport (GO:0006893), responsible for directed movement of substances from the Golgi to other parts of the cell and plasma membrane, growth factor receptor binding (GO:0070851) responsible for binding to a growth factor were also seen to be related to body conformation traits. These genomic regions contribute to the phenotypic expression of body conformation traits by regulating key biological process such as cellular differentiation, and structural organization, which are essential for overall body morphology in cattle.

#### 4.2.4 Expected progeny difference at weight 120

The expected Progeny Difference (EPD) for weight at 120 days reflects a sire’s genetic potential to produce offspring with superior/inferior weight at 120 days of age compared to that of progeny from other sires (Spangler, 2011; Bessin and Bullock, 2014). In practice, EPD are used to make a prediction of how the future offspring of an animal are anticipated to perform in comparison to the offspring of other animals recorded in the database, assuming the sires mated cows of similar genetic potential and the offspring are managed under similar environments (Bessin and Bullock, 2014; Spangler, 2011). EPD is an important selection tool in the beef industry, used for decades to estimate the genetic value of an animal as a parent (Bessin and Bullock, 2014). EPDs are expressed in the same unit that the trait was measured and can be positive or negative (Bessin and Bullock, 2014; NC State, 2023). The top 1% genomic windows were found in 14 chromosomes collectively accounting for 2.978% of additive genetic variance. These genomic regions encompass 239 genes as seen in Table 4.

**TABLE 4:** Candidate genes located within the top 1% genomic windows associated with expected progeny difference for weight at 120 days in Nellore cattle.

BTA	Position	Candidate genes	^1^Var (%)
14	27,356,198–28,167,750	*GGH, TTPA, YTHDF3, NKAIN3*	0.176
6	69,778,772–70,726,478	*KDR, KIT*	0.171
13	28,615,840–29,315,862	*FRMD4A, FAM107B*	0.168
11	48,548,918–50,040,146	*SH2D6, CAPG, ATOH8, ELMOD3, RETSAT, TCF7L1, KCMF1, REEP1, MRPL35, IMMT, PTCD3, POLR1A, ST3GAL5, SFTPB, USP39, C11H2orf68, RNF181, TMEM150A, VAMP8, GGCX, MAT2A, VAMP5*	0.161
28	37,957,840–38,948,561	*NRG3*	0.157
2	62,334,550–63,428,637	*CCNT2, ACMSD, TMEM163, MGAT5*	0.155
6	8,015,259–8,845,830	*TRAM1L1*	0.155
14	26,403,506–27,038,720	*CLVS1, ASPH, CHD7*	0.153
25	3,538,408–4,345,274	*CORO7, VASN, DNAJA3, NMRAL1, HMOX2, CDIP1, C16orf96, UBALD1, MGRN1, NUDT16L1, ANKS3, DNAAF8, ZNF500, SEPTIN12, ROGDI, GLYR1, UBN1, PPL, SEC14L5, NAGPA, C25H16orf89, ALG1, EEF2KMT*	0.150
9	43,483,890–44,256,176	*CRYBG1, ATG5, PRDM1*	0.150
3	925,689–1,727,273	*MPZL1, RCSD1, CREG1, CD247, POU2F1, STYXL2, GPA33*	0.146
11	69,551,444–70,200,016	*LBH, YPEL5, ALK*	0.145
7	55,928,192–56,837,004	*-*	0.142
22	33,115,385–35,314,908	*SUCLG2, TAFA1, KBTBD8, LRIG1, SLC25A26*	0.138
7	35,336,701–36,102,653	*-*	0.137
3	50,316,445–51,352,377	*MTF2, DIPK1A, RPL5, EVI5, GFI1, RPAP2, GLMN, C3H1orf146, EPHX4, BTBD8*	0.135
7	104,804,124–105,826,744	*-*	0.135
26	39,704,328–40,706,891	*PLPP4, TIAL1, BAG3, INPP5F, MCMBP, SEC23IP*	0.135
17	22,884,099–24,133,086	*PABPC4L*	0.134
29	4,736,334–6,190,309	*CHORDC1, NAALAD2, TRIM77, TRIM64, FOLH1B, NOX4*	0.134

Proportion of the trait variance explained by each window (%).

The candidate gene *TMEM163*, highlighted in the window 62.33–63.43 Mb of BTA 2, is a member of the solute carrier 30 (SLC30) family. The *TMEM163* is known to act as a zinc-binding protein that helps transports zinc in cells, thereby ensuring zinc homeostasis (critical, as abnormal zinc homeostasis can cause growth retardation; Cuajungco et al., 2021; Fukada et al., 2011). Another candidate gene, *CREG1*, found in the 0.9–1.72 Mb window of BTA3, is a gene involved in cellular growth, differentiation, and homeostasis regulation. The study performed by Song et al. (2024) suggested that *CREG*1 may also be a target for skeletal muscle regeneration, potentially impacting EPD at 120 days (Song et al., 2024). The gene *TMEM150A*, located at 48.55–50.04 Mb on BTA 11, plays an important role in homeostasis by regulating production of phosphatidylinositol (4,5)-bisphosphate [PI (4,5) P] through modifying the composition of phosphatidylinositol 4-kinase (PI4K) at the plasma membrane. Phosphatidylinositol 4-kinase (PI4K) is a lipid kinase that plays a crucial role in the synthesis of phosphatidylinositol (4,5)-bisphosphate, a phospholipid required in membrane-associated signaling functions (Borges-Araújo and Fernandes, 2020; Li et al., 2021). The gene *FRMD4A*, located at 28.6–29.3 Mb on BTA13 is seen to be involved in cell structure, transport and regulating cell polarity which is important in transmitting weight by playing an important role in the activation of ARF6 which modulates cell polarity in neurons (Fine et al., 2015).

Other candidate genes identified are associated with various cellular processes, such as transcription by RNA polymerase I (GO:0006360), cytolytic granule (GO:0044194), intracellular anatomical structure (GO:0005622), zinc ion binding (GO:0008270), ubiquitin-protein ligase (GO:0061630), ubiquitin-protein transferase (GO:0004842), and transition metal ion binding (GO:0046914). These genomic regions contribute to the phenotypic expression of this trait by regulating essential biological processes such as zinc homeostasis, and membrane signaling, ultimately influencing the sire’s genetic potential to produce offspring with superior/inferior weight at 120 days of age.

#### 4.2.5 Finishing score

Frame scoring, a method for measuring cattle skeletal size, reflects the potential mature size of an animal, and is used to project mature size and determine the potential and nutritional requirements of cattle (Dhuyvetter, 1995). The frame scoring system recommended by the Beef Improvement Federation (BIF) ranges from 1 to 10, with most scores ranging from two to 9 (Dhuyvetter, 1995). The top 1% genomic windows were spread across 14 chromosomes and collectively explained 3.008% of the additive genetic variance for the finishing score. These regions code 255 genes as seen in Table 5.

**TABLE 5:** Candidate genes located within the top 1% genomic windows associated with finishing score in Nellore cattle.

BTA	Position (BP)	Candidate genes	Var (%)
14	27,187,105–27,959,583	*ASPH, NKAIN3*	0.240
14	25,982,859–26,706,002	*CA8, RAB2A, CHD7*	0.176
21	50,708,665–51,628,186	*LRFN5*	0.163
10	59,283,823–60,764,907	*TNFAIP8L3, GABPB1, HDC, SLC27A2, AP4E1, ATP8B4, DTWD1, FAM227B, SPPL2A, TRPM7, USP50, USP8*	0.162
20	9,802,407–10,745,794	*BDP1, SERF1A, GTF2H2, TAF9, OCLN, MARVELD2, RAD17, AK6, CCDC125, CDK7, CENPH, CCNB1, SLC30A5, SMN2, NAIP, CARTPT, MCCC2*	0.157
6	69,343,468–70,257,540	*CHIC2, GSX2, PDGFRA, KIT*	0.157
6	32,912,211–33,991,326	*CCSER1*	0.154
23	28,398,875–29,002,896	*TRIM15, TRIM10, TRIM40, TRIM31, RNF39, PPP1R11, POLR1H, ZFP57, MOG, GABBR1, ABCF1, RPP21, GNL1, TRIM39, TRIM26, PRR3*	0.150
9	43,583,627–44,776,539	*PREP, CRYBG1, ATG5, PRDM1*	0.148
9	13,346,364–14,401,787	*-*	0.146
3	25,432,126–26,066,448	*TENT5C, VTCN1,MAN1A2*	0.144
8	32,479,887–33,601,105	*-*	0.142
20	61,807,939–62,244,291	*CTNND2*	0.135
13	60,763,905–61,948,082	*KIF3B, DEFB119, ASXL1, NOL4L, DEFB116, DEFB121, DEFB122A, DEFB122, DEFB123, DEFB124, REM1, HM13, ID1, COX4I2, HCK, TM9SF4, PLAGL2, POFUT1, BCL2L1, TPX2, MYLK2, FOXS1, DUSP15, TTLL9, PDRG1, DEFB129, XKR7, CCM2L, DEFB127, DEFB115*	0.135
2	69,283,654–71,244,759	*EN1, MARCO, C1QL2, STEAP3, C2H2orf76, DBI, TMEM37, SCTR, INSIG2, DDX18, CCDC93*	0.135
4	103,764,705–104,758,316	*SLC37A3, RAB19, MKRN1, DENND2A, ADCK2, NDUFB2, BRAF, TMEM178B, MRPS33*	0.135
2	105,100,249–106,205,451	*TNS1, RUFY4, CXCR2*	0.134
9	27,857,764–28,620,652	*SMPDL3A, FABP7, PKIB, TRDN, CLVS2*	0.132
11	79,606,327–80,652,125	*NT5C1B, RDH14*	0.132
11	46,688,746–47,677,419	*PSD4, PAX8, EIF2AK3, SPMIP9, FOXI3, RPIA, IL36A, IL36RN, IL1F10, IL1RN*	0.129

Proportion of the trait variance explained by each window (%).

A candidate gene, *FABP7*, located in the genomic window 27.86–28.62 Mb of BTA9, is a fatty acid-binding protein involved in fatty acid uptake, transportation, metabolism, and storage (Wang et al., 2023; George Warren et al., 2024). *FABP7* plays a role in regulating lipid metabolism and is involved in the proliferation of astrocytes by controlling cellular fatty acid homeostasis (Hamilton et al., 2023). Another candidate gene, *RDH14*, located in the genomic region 79.61–80.65 Mb of BTA11, is involved in osteoblast differentiation, a function directly related to the finishing score. For instance, osteoblasts are essential for skeletal development and require precise regulation during differentiation to ensure proper skeletal formation (Ponzetti and Rucci, 2021). The region between 60.7 and 61.9 Mb of BTA13 annotates multiple genes from the beta-defensin family (*DEFB123, DEFB124, DEFB127, DEFB115, DEFB116, DEFB121*), which possess cationic antimicrobial properties (Lehrer et al., 2007; Yamaguchi and Ouchi, 2012) and contribute to the innate immune response and reaction to external stimuli (Zhang et al., 2024). The candidate gene in region 27.18–27.96 Mb of BTA14 highlighted *ASPH* gene that encodes a protein that regulate the process of excitation-contraction in muscles associated to finishing score (Endo et al., 2022). Another highlighted gene, *LRFN5* (also known as SALM5), located in the window 50.7–51.6 Mb on BTA21, is involved in the regulation of neural and synaptic development and organization (Xu et al., 2023).

Other candidate genes identified are associated with immune response (GO:0006955), innate immune response (GO:0045087), response to external stimulus (GO:0009605), lumenal side of endoplasmic reticulum membrane (GO:0098553), growth factor receptor binding (GO:0070851), and biological process involved in interspecies interaction between organisms (GO:0044419). These genomic regions contribute to phenotypic variation of this trait by encoding genes that regulate key processes such as skeletal development, immune response, and muscle function, ultimately influencing the overall finishing potential of the cattle.

#### 4.2.6 Marbling

Marbling, defined as the appearance of evenly distributed white flecks or streaks of fatty tissue intermingled among muscle fibers, is an important trait determining meat quality and is one of the primary factors the consumers consider when buying meat (Kruk et al., 2002; Qiao et al., 2007; Cheng et al., 2015). Meat marbling is currently being evaluated by several techniques such as visual appraisal, chemical analysis, and other instrumental techniques to determine the degree of marbling depending on the standard of marbling evaluation in each country (Cheng et al., 2015). The top 1% genomic windows for this trait were spread in 13 chromosomes and collectively explained 2.995% of the additive genetic variance for marbling. These regions are responsible for coding 369 genes as seen in Table 6.

**TABLE 6:** Candidate genes located within the top 1% genomic windows associated with marbling in Nellore cattle.

BTA	Position (BP)	Candidate genes	^1^Var (%)
6	78,618,080–79,562,190		0.253
5	47,394,459–49,379,302	*HMGA2, MSRB3, LEMD3, WIF1, TBC1D30, GNS, RASSF3, TBK1, XPOT, C5H12orf56, GRIP1, HELB, IRAK3, TMBIM4, LLPH*	0.193
6	79,585,576–80,176,126	*TECRL*	0.178
7	71,104,335–71,828,825	*ADRA1B, TTC1, PWWP2A, CCNJL, FABP6*	0.167
2	125,434,119–127,293,219	*AHDC1, WASF2, GPR3, CD164L2, MAP3K6, SYTL1, TMEM222, WDTC1, SLC9A1, TENT5B, TRNP1, KDF1, NUDC, NR0B2, FGR, GPATCH3, GPN2, SFN, ZDHHC18, PIGV, ARID1A, RPS6KA1, HMGN2, DHDDS, LIN28A, ZNF683, CRYBG2, CD52, UBXN11, SH3BGRL3, CEP85, CATSPER4, CNKSR1, FAM110D, ZNF593, C1orf232, PDIK1L, TRIM63, SLC30A2, EXTL1, PAFAH2, RPA2, THEMIS2, STMN1, PAQR7, MTFR1L, SELENON, PPP1R8, STX12, MAN1C1, FAM76A, IFI6*	0.160
14	23,402,733–24,474,202	*SDR16C5, SDR16C6, PENK, BPNT2, FAM110B*	0.152
27	9,400,186–10,347,573	*-*	0.151
2	27,761,184–28,585,153	*STK39, B3GALT1*	0.147
5	55,473,558–58,186,804	*HSD17B6, PRIM1, NACA, PTGES3, ATP5F1B, BAZ2A, RBMS2, GLS2, SPRYD4, MIP, TIMELESS, APON, APOF, STAT2, IL23A, PAN2, CNPY2, COQ10A, ANKRD52, SLC39A5, NABP2, RNF41, SMARCC2, MYL6B, ESYT1, ZC3H10, PA2G4, ERBB3, RPS26, IKZF4, SUOX, RAB5B, CDK2, PMEL, DGKA, PYM1, MMP19, TMEM198B, DNAJC14, ORMDL2, GDF11, CD63, RDH5, ITGA7, TMT1B, ATP23, CTDSP2, AVIL, TSFM, EEF1AKMT3, METTL1, CYP27B1, MARCHF9, CDK4, TSPAN31, AGAP2, OS9, B4GALNT1, SLC26A10, ARHGEF25, DTX3, PIP4K2C, KIF5A, DCTN2, MBD6, DDIT3, MARS1, ARHGAP9, GLI1, INHBE, INHBC* *R3HDM2, STAC3, NDUFA4L2, SHMT2, NXPH4, LRP1, STAT6, NAB2, NEMP1, MYO1A, TAC3, ZBTB39, GPR182, RDH16, SDR9C7, OR6C17, OR10P25, OR10P1, OR6C63, OR6C304, OR6C8, OR6C267, OR6C4, OR6C288, OR6C264, OR6C22, OR6C4C, OR6C38, OR6C2F, OR6C277, OR6C202, OR6C278, CS, MYL6, SARNP, BLOC1S1*	0.140
5	12,374,029–13,049,667	*TMTC2*	0.137
22	18,808,954–19,706,570	*GRM7*	0.136
8	81,874,543–82,796,028	*FANCC, PTCH1, ERCC6L2*	0.136
14	79,397,442–80,096,793	*-*	0.134
23	48,783,733–49,358,228	*FARS2, NRN1, F13A1*	0.133
1	145,647,961–146,394,034	*PRMT2, COL6A1, COL6A2, FTCD, SPATC1L, LSS, MCM3AP, YBEY, PCNT, DIP2A, S100B*	0.133
17	44,107,538–44,886,429	*ZNF140, ZNF10, ZNF268, MBD3L1, ANHX, CHFR, GOLGA3, ANKLE2, PGAM5, POLE, P2RX2, LRCOL1, FBRSL1, PXMP2, GALNT9*	0.133
14	4,426,123–5,104,567	*FAM135B*	0.129
9	35,156,452–35,891,042	*-*	0.128
8	49,642,335–50,652,903	*-*	0.128
12	22,998,566–23,954,600	*LHFPL6, NHLRC3, PROSER1, STOML3, FREM2, UFM1*	0.128

Proportion of the trait variance explained by each window (%).

Arikawa et al. (2024) performed genome-wide association studies for meat quality traits and identified a window in BTA7 (64.6–65.6 Mb) close to the region identified in our study (71.1–71.8 Mb on BTA7), also explaining the largest variance for marbling (Arikawa et al., 2024). The window reported for BTA7 in their study includes the genes *PWWP2A* and *FABP6*, which are known to be responsible for enabling chromatin binding, bile acid binding, and histone binding activity–functions that are crucial for high-quality marbling in beef cattle (Hendrick et al., 2016; Link et al., 2018). In addition, the region 22.9–23.9 Mb of BTA12, identified in our study, highlights *PROSER1,* a probable novel gene that regulates chromatin (required in muscling) association of TET, a key player in DNA methylation. This regulation is essential for muscle development, as excessive demethylation can cause developmental malformations (Wang et al., 2021).

Interestingly, Carvalho et al. (2019) identified a genomic window at 70.77–70.78 Mb on BTA6 associated with marbling, while our study pinpointed a different region on BTA6, from 78.62 to 79.56 Mb (Carvalho et al., 2019). This region (in our study) includes *TECRL*, a protein localized in the endoplasmic reticulum and specifically expressed in heart and skeletal muscle tissues (Geng et al., 2024), suggesting potential functional relevance in marbling. Additionally, the candidate gene *SELENON* was identified in the window 125.43–127.73 Mb of BTA2. Thus, the gene localized in the endoplasmic reticulum provides instructions for making selenoprotein, which are involved in oxidation-reduction activities essential for protecting cells from oxidative stress (Chernorudskiy et al., 2020). This gene is very important in marbling, as the mutation in selenoprotein, as shown by the study performed by Bouman et al. (2021), can cause selenoprotein-related myopathy, a rare congenital myopathy that causes muscle weakness (Bouman et al., 2021). Other GO terms identified include the terpenoid metabolic process (GO:0006721), diterpenoid metabolic process (GO:0016101), and isoprenoid metabolic process (GO:0006720), which is known to have anti-inflammatory effect which can be beneficial to the muscle as it promotes transdermal absorption (Mohammadi-Cheraghabadi and Hazrati, 2023). These genomic regions contribute to the phenotypic expression of marbling by encoding genes that regulate key processes such as chromatin modification, muscle development, and oxidative stress response, ultimately influencing intramuscular fat deposition and meat quality in cattle.

#### 4.2.7 Muscularity

Muscling, defined as the degree of thickness relative to an animal’s frame size, is typically assessed using the muscle score (McKiernan, 2017). The top 1% genomic windows associated with muscularity were spread in 15 chromosomes and collectively explained 3.013% of the additive genetic variance for muscularity. These regions are responsible for coding 203 genes as seen in Table 7. Similarly, the maximum additive genetic variance explained by 1 MB window of adjacent SNPs (population genotyped using the 777 K SNP panel) was approximately 0.4% in Carreño et al. (2019) for muscularity (Carreño et al., 2019).

**TABLE 7:** Candidate genes located within the top 1% genetic windows associated with muscularity in Nellore cattle.

BTA	Position (BP)	Candidate genes	^1^Var (%)
6	69,261,988–70,155,060	*CHIC2, GSX2, PDGFRA*	0.232
16	60,596,486–61,534,166	*SOAT1, AXDND1, NPHS2, TDRD5, FAM163A, TOR1AIP2, TOR1AIP1, CEP350, QSOX1, LHX4, ACBD6*	0.193
3	56,477,441–57,243,927	*LMO4, HS2ST1, SELENOF*	0.173
8	25,904,409–27,055,794	*ADAMTSL1, SH3GL2, CNTLN*	0.169
10	85,381,223–86,410,610	*BBOF1, ALDH6A1, LIN52, VSX2, ABCD4, VRTN, SYNDIG1L, NPC2, ISCA2, LTBP2, AREL1, FCF1, YLPM1, PROX2, DLST, RPS6KL1, PGF, EIF2B2, ACYP1, ZC2HC1C, NEK9, TMED10, MLH3*	0.168
9	80,031,480–80,701,610	*HIVEP2, AIG1*	0.167
28	38,520,118–39,377,854	*GHITM, GPR15LG, CDHR1, LRIT2, LRIT1, RGR, CCSER2*	0.166
5	40,610,629–41,720,384	*SLC2A13, C12orf40, ABCD2, LRRK2*	0.150
10	82,639,946–83,444,113	*SIPA1L1, PCNX1*	0.142
14	9,011,203–9,987,316	*KCNQ3, HHLA1, OC90, EFR3A, ADCY8*	0.142
27	22,145,571–23,213,576	*SGCZ*	0.138
23	11,542,777–13,065,602	*ZFAND3, BTBD9, GLO1, DNAH8, GLP1R, SAYSD1, KCNK5, MDGA1*	0.136
15	57,685,620–58,434,538	*FIBIN, BBOX1, CCDC34, LGR4, LIN7C, BDNF*	0.135
14	4,003,143–4,546,888	*COL22A1, FAM135B*	0.134
14	41,537.492–42,576,327	*PKIA, IL7*	0.131
11	90,288,247–91,116,303	*SOX11*	0.129
8	80,792,014–81,428,622	*DAPK1, CTSL, FBP2, AOPEP, FBP1*	0.129
13	23,941,098–24.857,890	*ARMC3, MSRB2, PTF1A, OTUD1*	0.128
20	29,800,340–31,105,010	*MRPS30, FGF10*	0.127
3	25,473,297–26,095,766	*TENT5C, VTCN1, MAN1A2*	0.126

Proportion of the trait variance explained by each window (%).

The study performed by Machado et al. (2022) identified genomic regions associated with muscularity, and highlighted windows located on BTA9 and BTA16, which were consistent with the findings from our study (Machado et al., 2022). In addition, the BTA3, BTA5, BTA20, and BTA23 highlighted in the study by Carreño et al. (2019) on candidate genes for muscling traits, also overlapped with our findings (Carreño et al., 2019). One noteworthy gene, *FGF10*, located in the genomic window 29.8–31.1 MB of BTA20, plays a critical role in maintaining tissue homeostasis, myogenesis and coordinating alveolar smooth muscle cell formation (Zhang et al., 2006; Nurgulsim et al., 2023). Disruptions in *FGF10* are linked to defects in limb development and organ branching (Bellusci et al., 1997; Arman et al., 1999; Sekine et al., 1999). A likely novel candidate gene, *HS2ST1*, was found in the genomic region 56.4–57.2 Mb on BTA3, is involved in cell signaling and development and has a known role in fibroblast growth factor binding (Schneeberger et al., 2020).

The *ADAMTSL1* gene, also known as Punctin, was identified in the genomic window 25.9–27.0 Mb on BTA8. As a member of the ADAMTS family, ADAMTSL1 is implicated in muscle function, particularly in binding extracellular matrix substrates (Kuno et al., 1999; Hirohata et al., 2002). Additionally, it shows potential as a treatment target for muscular dystrophy (Du et al., 2017; Wang et al., 2021). Several other candidate genes identified are associated with GO terms like metanephric glomerulus development (GO:0072224), and fructose 1,6-bisphosphate 1-phosphatase activity (GO:0042132). These genomic regions contribute to the phenotypic expression of muscularity by encoding genes that regulate the key biological processes such as extracellular matrix organization, muscle cell development, and fibroblast growth factor, which enhances muscle growth and overall meat quality in cattle.

#### 4.2.8 Ribeye area

Ribeye area is an important trait to evaluate carcass quality (Zhao et al., 2022) and holds significant value for cattle producers by helping to determine production efficiency, beef yield, and economic return (Goodall and Schmutz, 2007; Meirelles et al., 2011). The top 1% genomic windows were spread across 13 chromosomes and jointly explained 2.962% of the additive genetic variance for the ribeye area. These regions are responsible for coding 238 genes as seen in Table 9. Interestingly, the additive genetic variance explained for the ribeye area ranged from 0.71% to 1.24% in the study performed by Arikawa et al. (2024), which differs from the maximum additive genetic variance of 0.186% found in our study. This difference can likely be attributed to factors such as the Nellore population, differences in the statistical model, and the window type and size used for estimating the proportion of variance explained by the SNPs (Arikawa et al., 2024).

Santana et al. (2015) identified candidate genes associated with ultrasound-derived measurements of the ribeye area, specifically at BTA1, BTA3, BTA4, BTA8, BTA20, and BTA21. While these chromosomes were also detected in our study, the associated windows differed. For instance, the window 53.1–53.7 Mb of BTA 3, highlights the *LRRC8D* gene, which facilitates the permeation of organic substrates (Nakamura et al., 2020). The window located on 13.8–14.5 Mb of BTA21 is related to *CHD2*, a gene that plays a vital role in chromatin structure (Lewis et al., 2022). Interestingly, Santana et al. (2015) also highlighted a window on BTA20 without gene annotation (Santana et al., 2015), while our study identified a nearby window (29.8–31.09 Mb), which includes *FGF10*, a gene essential for tissue homeostasis and alveolar smooth muscle formation (Zhang et al., 2006).

Arikawa et al. (2024) found regions at 115.7 Mb on BTA3 and 79.2 Mb on BTA9 associated with the ribeye area (Arikawa et al., 2024). In contrast, our study identified the window 100.49–101.49 Mb on BTA3 and 79.98–80.64 Mb on BTA9. Notably, *HIVEP2*, a transcription factor involved in neural development, was annotated in both the 79.2 Mb region of BTA9 from Arikawa et al. (2024) and our BTA9 window (Park et al., 2023; Arikawa et al., 2024). Reis et al. (2023) also identified BTA1, BTA8, BTA9, and BTA11 as linked to the ribeye area, with different windows highlighted in our study (Reis et al., 2023). For example, the gene *ROBO2* at 22.88–23.66 Mb on BTA1 acts as a stroma suppressor gene by restraining myofibroblast activation and T-cell infiltration (Pinho et al., 2018). Additionally, *ADAMTSL1*, annotated at 25.9–27.1 Mb on BTA8 for ribeye area, plays a role in muscle function and binds to extracellular matrix substrates (Kuno et al., 1999; Hirohata et al., 2002). The window 57.83–58.92 Mb on BTA10 includes two myosin superfamily genes, *MYO5A* and *MYO5C*, both of which are involved in actin-based cellular functions (Jacobs et al., 2009; Carew et al., 2023).

Other candidate genes identified in our study are associated to volume-sensitive anion channel activity (GO:0005225), and Spliceosome (KEGG:03,040). The identified genomic regions contribute to the phenotypic expression of ribeye area as the encoding genes in these regions were seen to regulate key biological processes such as chromatin remodeling and extracellular matrix organization, ultimately influencing the carcass composition in cattle.

#### 4.2.9 Finishing precocity

Finishing precocity trait is an important trait used to estimate the time it takes an animal to reach slaughter from birth (Everling et al., 2014). The top 1% of genomic windows were spread across 13 chromosomes (BTA1, BTA 3, BTA 6, BTA 7, BTA 8, BTA 9, BTA 10, BTA 14, BTA 16, BTA 20, BTA 21, BTA 23, BTA 27), and jointly explained 3.158% of the additive genetic variance for finishing precocity. These genomic regions are responsible for coding 217 genes as seen in Table 8. Notably the maximum additive genetic variance explained of approximately 0.40% associated with finishing precocity in the study by Carreño et al. (2019) seems to be comparable to the maximum additive genetic variance of 0.243% identified for finishing precocity in our study.

**TABLE 8:** Candidate genes located within the top 1% genomic windows associated with finishing precocity in Nellore cattle.

BTA	Position (BP)	Candidate genes	^1^Var (%)
6	69,261,988–70,155,060	*CHIC2, GSX2, PDGFRA*	0.243
10	85,381,223–86,410,610	*BBOF1, ALDH6A1, LIN52, VSX2, ABCD4, VRTN, SYNDIG1L, NPC2, ISCA2, LTBP2, AREL1, FCF1, YLPM1, PROX2, DLST, RPS6KL1, PGF, EIF2B2, ACYP1, ZC2HC1C, NEK9, TMED10, MLH3*	0.233
8	25,904,409–27,055,794	*ADAMTSL1, SH3GL2, CNTLN*	0.194
9	80,031,480–80,701,610	*HIVEP2, AIG1*	0.167
16	60,596,486–61,534,166	*SOAT1, AXDND1, NPHS2, TDRD5, FAM163A, TOR1AIP2, TOR1AIP1, CEP350, QSOX1, LHX4, ACBD6*	0.166
3	56,477,441–57,243,927	*LMO4, HS2ST1, SELENOF*	0.163
1	127,925,159–128,739,175	*SLC25A36, TRIM42, CLSTN2*	0.161
3	95,993,334–97,202,518	*ELAVL4*	0.155
14	9,099,016–10,055,338	*OC90, EFR3A, ADCY8*	0.151
27	9,569,573–10,535,908	*-*	0.150
20	29,812,331–31,112,179	*MRPS30, FGF10*	0.150
7	37,262,036–38,807,831	*UIMC1, NSD1, RAB24, PRELID1, MXD3, ZNF346, FGFR4, COMMD10, SEMA6A, UNC5A, HK3, FAF2, RNF44, CDHR2, SNCB, EIF4E1B, TSPAN17, ARL10, NOP16, HIGD2A, CLTB, GPRIN1*	0.149
1	3,954,394–4,499,092	*TIAM1*	0.144
3	25,262,583–25,968,219	*WDR3, GDAP2, TENT5C, MAN1A2*	0.141
10	82,639,946–83,444,113	*SIPA1L1, PCNX1*	0.133
23	11,558,107–13,089,738	*ZFAND3, BTBD9, GLO1, DNAH8, GLP1R, SAYSD1, KCNK5, MDGA1*	0.133
21	35,060,169–35,613,940	*STXBP6*	0.132
14	41,543,268–42,586,262	*PKIA, IL7*	0.132
8	57,399,938–58,531,522	*TLE1*	0.132
6	28,927,865–29,651,751	*UNC5C, BMPR1B*	0.130

Proportion of the trait variance explained by each window (%).

**TABLE 9:** Candidate genes located within the top 1% genomic windows associated with ribeye area in Nellore cattle.

BTA	Position (BP)	Candidate genes	^1^Var (%)
1	22,881,541–23,663,115	*ABCC13, RBM11, LIPI, ROBO2*	0.186
3	53,158,842–53,791,569	*LRRC8D, LRRC8C, LRRC8B, ZNF326*	0.182
6	69,286,821–70215,009	*CHIC2, GSX2, PDGFRA, KIT*	0.180
20	29,797,692–31,090,381	*MRPS30, FGF10*	0.178
3	100,493,684–101,486,056	*AKR1A1, PRDX1, MMACHC, CCDC163, TESK2, TOE1, MUTYH, HPDL, ZSWIM5, UROD, HECTD3, KIF2C, ARMH1, TMEM53, EIF2B3, PTCH2, DYNLT4, BTBD19, PLK3, BEST4, RPS8, RNF220*	0.170
10	82,605,716–83,416,877	*SIPA1L1, PCNX1*	0.161
9	79,980,066–80637,950	*HIVEP2, AIG1*	0.155
9	39,631,280–4,0212,035	*CDK19, SLC22A16, DDO, METTL24, CDC40, WASF1*	0.150
23	11,558,107–13,089,738	*ZFAND3, BTBD9, GLO1, DNAH8, GLP1R, SAYSD1, KCNK5, MDGA1*	0.149
3	67,459,768–68,486,051	*PIGK,ST6GALNAC5, ST6GALNAC3*	0.141
3	5,391,773–6221,900	*NUF2, RGS5*	0.139
21	13,829,973–14,576,562	*RGMA, ST8SIA2, FAM174B, CHD2*	0.137
14	41,589,449–42,600,138	*PKIA, IL7*	0.135
1	114,032,073–114,997,565	*P2RY1, RAP2B*	0.134
4	62,848,985–64,673,068	*PDE1C, BMPER, BBS9, RP9, NT5C3A, FKBP9, KBTBD2, AVL9, LSM5*	0.131
8	25,904,409–27,055,794	*ADAMTSL1,SH3GL2, CNTLN*	0.130
15	57,651,202–58,404,974	*FIBIN, BBOX1, CCDC34, LGR4, LIN7C, BDNF*	0.128
11	5,791,339–6390,618	*CNOT11, RNF149, NPAS2, RPL31, TBC1D8, RFX8, CREG2*	0.127
14	36,637,187–37,728,473	*STAU2, UBE2W, ELOC, TMEM70, LY96, JPH1, GDAP1, RDH10*	0.125
10	57,834,717–58,921,372	*MYO5A, MYO5C, GNB5, MAPK6, LEO1, TMOD3, TMOD2, LYSMD2, SCG3, DMXL2*	0.125

Proportion of the trait variance explained by each window (%).

A study performed by Carreño et al. (2019), also identified the BTA16 and BTA23 as chromosomes associated with finishing precocity, although different genomic locations were identified in our study (Carreño et al., 2019). The study by Machado et al. (2022) identified the genomic region 11.56–12.44 Mb located on BTA 23 (Machado et al., 2022), as a region associated with precocity traits. Interestingly, this region was also found in our study, i.e., 11.56–13.09 Mb on BTA 23. This region was significant for both finishing precocity, muscularity, and ribeye area, and contained eight genes common across these traits, including *ZFAND3, BTBD9, GLO1, DNAH8, GLP1R, SAYSD1, KCNK5,* and *MDGA*1. Of these, *ZFAND3* enables DNA binding and zinc ion binding activity, supporting findings by Hlongwane et al. (2020), who reported associations with meat and growth traits in pigs (Hlongwane et al., 2020).

The genomic window 60.5–61.5 Mb on BTA16 highlights the *SOAT1* gene, which plays a role in lipoprotein and cholesterol absorption, crucial for maintaining membrane microstructures (Tu et al., 2023). Another genomic region, 25.2–25.9 Mb on BTA3, features the *MAN1A2* gene. In a study by Flanigan et al. (2021) on Duchenne muscular dystrophy, MAN1A2 was found to be important for muscle ion channel function, cell adhesion, and muscle stem cell function (Flanigan et al., 2021). Reducing MAN1A2 expression, which involves the removal of sialic acid, was shown to alleviate muscular dystrophy symptoms, suggesting its potential importance in finishing precocity (Flanigan et al., 2021).

Additional candidate genes identified in this study are related to cellular component organization (GO:0016043) and Rap1 signaling pathway (KEGG:04,015), which regulates responses to external stimuli, controls processes such as cell adhesion, cell-cell junction formation, and cell polarity which are involved in cytoskeleton dynamics (Hilbi and Kortholt, 2019). These genomic regions contribute to the phenotypic expression of the finishing precocity traits as the encoding genes in these regions were seen to regulate key biological processes such as lipid metabolism, and cellular signaling, ultimately enhancing the animals ability to reach market weight.

#### 4.2.10 Scrotal circumference at 365days and 450 days of age

Scrotal circumference is an important selection criterion for selecting bulls, as it is correlated with daily sperm production (Soren, 2021), and it is a useful predictor of age at puberty, and has high heritability (Knights et al., 1984). Improved scrotal circumference is beneficial for the reproductive performance in the beef cattle industry (Menegassi et al., 2019). The top 1% genomic windows were spread in 14 chromosomes for SC365, and 15 chromosomes for SC450. Interestingly, the top 1% windows jointly explained 3.005% of the additive genetic variance for both SC365 and SC450. However, the genomic regions related to SC365 are responsible for codifying 268 genes (Table 10., Supplementary Material S10), while the top genomic regions related to SC450 are responsible for codifying 272 genes (Table 11., Supplementary Table S1). Although many of the same genomic windows are annotated at both ages, BTA2, BTA3, and BTA20 are exclusive to SC365, while BTA15 and BTA25 are exclusive to SC450. The additive genetic variance explained for SC365 and SC450 ranged from 0.1% to 0.84%, and from 0.1% to 2.78%, respectively, in the study performed by Sbardella et al. (2021).

**TABLE 10:** Candidate genes located within the top 1% genomic windows associated with scrotal circumference at 365days in Nellore cattle.

BTA	Position (BP)	Candidate genes	^1^Var (%)
6	69,356,875–70295,716	*CHIC2, GSX2, PDGFRA, KIT*	0.197
10	82,639,946–83,444,113	*SIPA1L1, PCNX1*	0.193
23	11,550,429–13,086,890	*ZFAND3, BTBD9, GLO1, DNAH8, GLP1R, SAYSD1, KCNK5, MDGA1*	0.166
7	40,783,586–41,958,721	*TRIM58, OR2W3, GCSAML, OR2AJ9, OR2L2B, OR2L2C, OR2L3C, OR2L13, OR2T22, OR2M16, OR2M4, OR9E2, OR5AE4, OR5AE3, OR6F1, OR2AK3, OR2T62, OR2T63, OR2T16, OR2T60, OR6AA1, OR6AN1, OR11L1, OR2L3, OR14P2, OR2L2, OR2AO1, OR2T54, OR2W3D, OR2W53, OR2C3, OR2C3B, OR2G27, OR2G28, OR2G3*	0.166
1	116,725,563–117,854,869	*MED12L, P2RY12, P2RY13, GPR87, P2RY14, GPR171, CLRN1, MINDY4B, SIAH2, ERICH6, SELENOT, EIF2A, SERP1, TSC22D2*	0.161
28	8,872,423–9797,980	*EDARADD, LGALS8, HEATR1, ACTN2, MTR, RYR2, GPR137B, ERO1B*	0.159
20	29,753,913–31,057,622	*MRPS30, FGF10*	0.157
2	112,874,857–113,544,553	*DOCK10, NYAP2*	0.154
3	50,416,005–5,1,431,993	*DIPK1A, RPL5, EVI5, GFI1, RPAP2, GLMN, C3H1orf146, EPHX4, BRDT, BTBD8*	0.150
9	43,539,013–44,445,325	*CRYBG1, ATG5, PRDM1*	0.149
23	1,419,021–2439,750	*-*	0.142
7	39,069,981–39,942,634	*RMND5B, NHP2, HNRNPAB, PHYKPL, B4GALT7, N4BP3, TMED9, COL23A1, CLK4, ZNF354A, PROP1, OR7A129*	0.141
21	35,060,169–35,613,940	*STXBP6*	0.137
9	87,926,162–88,656,403	*CCDC170, PLEKHG1, MTHFD1L, ZBTB2, RMND1, ARMT1, AKAP12*	0.136
5	9,302,435–10500,596	*MYF6, MYF5, LIN7A, PPP1R12A, OTOGL, PTPRQ*	0.136
8	67,228,816–68,010,954	*SLC18A1, ATP6V1B2, LZTS1*	0.135
8	103,366,368–104,185,685	*ATP6V1G1, TMEM268, TNFSF15, TNFSF8, AKNA, WHRN, COL27A1, ORM1*	0.133
10	85,072,909–85,955,384	*PNMA1, MIDEAS, PTGR2, ZNF410, FAM161B, COQ6, ENTPD5, BBOF1, ALDH6A1, LIN52, VSX2, ABCD4, VRTN, SYNDIG1L, NPC2, ISCA2, LTBP2, AREL1, DNAL1*	0.132
8	34,236,056–35,005,404	*-*	0.131
27	37,477,404–38,273,045	*CHRNB3, CHRNA6, THAP1, RNF170, HOOK3, FNTA, POMK, HGSNAT, INTS10, CSGALNACT1, SH2D4A*	0.130

Proportion of the trait variance explained by each window (%).

**TABLE 11:** Candidate genes located within the top 1% genomic windows associated with scrotal circumference at 450 days in Nellore cattle.

BTA	Position (BP)	Candidate genes	^1^Var (%)
10	82,639,946–83,444,113	*SIPA1L1, PCNX1*	0.183
8	57,399,938–58,531,522	*TLE1*	0.159
6	69,283,831–70211,678	*CHIC2, GSX2, PDGFRA, KIT*	0.159
21	35,060,169–35,613,940	*STXBP6*	0.154
28	8,749,119–9703,912	*EDARADD, LGALS8, HEATR1, ACTN2, MTR, NID1, RYR2, GPR137B, ERO1B*	0.152
7	55,928,192–56,837,004	*-*	0.151
10	85,072,909–85,955,384	*PNMA1, MIDEAS, PTGR2, ZNF410, FAM161B, COQ6, ENTPD5, BBOF1, ALDH6A1, LIN52, VSX2, ABCD4, VRTN, SYNDIG1L, NPC2, ISCA2, LTBP2, AREL1, DNAL1*	0.149
7	40,783,586–41,958,721	*TRIM58, OR2W3, GCSAML, OR2AJ9, OR2L2B, OR2L2C, OR2L3C, OR2L13, OR2T22, OR2M16, OR2M4, OR9E2, OR5AE4, OR5AE3, OR6F1, OR2AK3, OR2T62, OR2T63, OR2T16, OR2T60, OR6AA1, OR6AN1, OR11L1, OR2L3, OR14P2, OR2L2, OR2AO1, OR2T54, OR2W3D, OR2W53, OR2C3, OR2C3B, OR2G27, OR2G28, OR2G3*	0.149
5	9,302,435–10500,596	*MYF6, MYF5, LIN7A, PPP1R12A, OTOGL, PTPRQ*	0.144
8	34,236,056–35,005,404	*-*	0.142
15	57,675,730–58,418,857	*FIBIN, BBOX1, CCDC34, LGR4, LIN7C, BDNF*	0.141
23	11,550,429–13,086,890	*ZFAND3, BTBD9, GLO1, DNAH8, GLP1R, SAYSD1, KCNK5, MDGA1*	0.140
23	1,419,021–2439,750	*-*	0.137
8	103,366,368–104,185,685	*ATP6V1G1, TMEM268, TNFSF15, TNFSF8, AKNA, WHRN, COL27A1, ORM1*	0.135
25	22,300,037–23,104,738	*ZKSCAN2, TNRC6A, SLC5A11, ARHGAP17, LCMT1, AQP8*	0.132
9	43,539,013–44,445,325	*CRYBG1, ATG5, PRDM1*	0.132
27	9,383,429–10346,575	*-*	0.132
21	58,457,450–59,106,537	*ASB2, CCDC197, OTUB2, DDX24, ISG12(B), IFI27, FAM181A, IFI27L2, PPP4R4, SERPINA10, SERPINA6, SERPINA1*	0.130
1	116,725,563–117,854,869	*MED12L, P2RY12, P2RY13, GPR87, P2RY14, GPR171, CLRN1, MINDY4B, SIAH2, ERICH6, SELENOT, EIF2A, SERP1, TSC22D2*	0.129
8	94,078,634–95,205,174	*NIPSNAP3A, ABCA1, SLC44A1, OR13F1, OR13C2E, OR13C2D, OR13F1B, OR13C2, OR13D2, OR13C2C, OR13D2C, OR13C8*	0.126

Proportion of the trait variance explained by each window (%).

Our findings align with previous studies that identified chromosomes associated with scrotal circumference, though with different windows. For instance, Irano et al. (2016) identified BTA8 and BTA23; Utsunomiya et al. (2014) identified BTA6, BTA10, and BTA 21; and Sbardella et al. (2021) identified BTA7, and BTA27 for SC365, and BTA5 and BTA23 for SC450, supporting the association of these chromosomes with scrotal circumference (Irano et al., 2016; Sbardella et al., 2021; Utsunomiya et al., 2014). One of the candidate genes highlighted in our study located in the window 50.4–51.4 Mb of BTA 3, the *EVI5* gene, is highlighted to be a novel centrosomal protein involved in centrosome stability, a vital organelle that plays a key role in fertilization and early embryonic development (Faitar et al., 2005). Another candidate gene, *BRDT*, identified in the window 50.4–51.4 Mb at BTA3 in our study, regulates meiotic division and it is essential for male germ cell differentiation (Shang et al., 2007). The probable novel gene *KIT,* annotated in the window 69.4–70.3 Mb located at BTA6, which has been known to be associated with coat color (Fontanesi et al., 2010) has also been associated with the male germ cell (Nakai et al., 2005). The candidate gene *CCDC34* annotated for SC450 in the window 57.6–58.4 Mb of BTA15, is involved in spermatogenesis and intraflagellar transport, which is very important for the formation of sperm flagella (Cong et al., 2022). A study performed by Rasool et al. (2023) showed that a loss in the candidate gene *LCMT*1 is related to prostate cancer (Rasool et al., 2023). Other genes candidate genes linked to SC365 and SC450 are involved in sensory perception (GO:0050906), detection of chemical stimulus GO:0009593, biological regulation GO:0065007, regulation of cellular process (GO:0050794), and regulation of biological process (GO:0050789). These genomic regions contribute to the phenotypic expression of scrotal circumference for age at 365 and 450days by encoding genes that regulate the key biological processes such as germ cell differentiation, sperm flagella formation and centrosome stability, which are essential for male fertility, and reproductive efficiency in cattle.

#### 4.2.11 Stayability

Stayability was defined by Hudson and Van Vleck (1981) as the ability of a cow to remain in the herd until a specific age, similar to longevity in dairy cattle (Hudson and Van Vleck, 1981). Stayability is a reproductive trait related to the cow’s ability to produce a certain number of calves over a given period, and it plays a major role in the overall profitability of the beef cattle industry (Buzanskas et al., 2010; Ramos et al., 2020). The top 1% genomic widows for stayability were spread in 11 chromosomes which jointly explained 2.779% of the additive genetic variance for stayability. These genomic regions are responsible for coding 216 genes as seen in Table 12. Notably the additive genetic variance explained for STAY ranged from 0.05% to 2.40% in the study performed by Sbardella et al. (2021).

**TABLE 12:** Candidate genes located within the top 1% genomic windows associated with stayability in Nellore cattle.

BTA	Position (BP)	Candidate genes	^1^Var (%)
5	78,607,994–79,944,005	*TMTC1, SINHCAF, CAPRIN2, IPO8*	0.164
23	24,526,787–24,904,607	*EFHC1, TRAM2, IL17A, IL17F,MCM3,PAQR8*	0.162
14	3,3,091,989–33,880,130	*PRDM14, NCOA2, SLCO5A1, SULF1*	0.153
23	30,895,043–31,650,938	*H2BC15, H2AC14, H1-5, ZNF391, PRSS16, H2BC11, ZNF322, ABT1, HMGN4, BTN1A1, BTN2A2, OR2B2D*	0.149
9	67,833,164–69,283,710	*ARHGAP18, TMEM244, L3MBTL3, SAMD3, TMEM200A, SMLR1, EPB41L2, AKAP7*	0.147
17	21,573,140–22,618,247	*-*	0.147
9	71,985,266–72,925,757	*SGK1, TCF21, TBPL1, SLC2A12*	0.147
5	75,925,896–77,024,658	*ALG10, SYT10, PKP2, CARD10, USP18*	0.141
6	73,353,617–74,233,590	*-*	0.140
18	16,130,241–16,971,062	*SIAH1, N4BP1, ABCC12, ABCC11, LONP2*	0.137
16	20,711,754–21,471,499	*GPATCH2, SPATA17*	0.136
18	37,992,979–40242,188	*ZFHX3, PMFBP1, DHX38, TXNL4B, HP, DHODH, PKD1L3, IST1, ZNF821, ATXN1L, AP1G1, PHLPP2, MARVELD3, TAT, CHST4, ZNF19, ZNF23, TLE7, CMTR2, CALB2, POP4*	0.135
23	3,4,391,772–35,702,735	*PRP1, GHE4, PRP9,PRP-VII,PRP3,CSH2,PRL,HDGFL1*	0.134
4	42,001,110–4,3,481,949	*MAGI2, PHTF2, TMEM60, RSBN1L*	0.132
15	34,513,822–34,997,350	*SERGEF, KCNC1, MYOD1, OTOG, USH1C*	0.131
29	3,1,041,924–32,854,344	*ETS1, BARX2, KCNJ5,FLI1,ARHGAP32,KCNJ1*	0.130
6	94,115,285–94,845,910	*ANTXR2, GK2*	0.124
14	63,437,379–64,320,590	*PABPC1, SNX31, ANKRD46, RNF19A, SPAG1, POLR2K, FBXO43, RGS22*	0.124
14	52,760,875–54,016,467	*-*	0.123
5	69,094,374–70688,878	*NUAK1, CKAP4, TCP11L2, POLR3B, RFX4, RIC8B, TMEM263, MTERF2, CRY1, BTBD11*	0.123

Proportion of the trait variance explained by each window (%).

The study by Sbardella et al. (2021), which aimed to identify candidate genes and biological pathways associated with stayability in Nellore cattle, highlighted BTA4, BTA9, and BTA15, which were also highlighted in our study (Sbardella et al., 2021). For instance, the SNP located on 43.3 Mb of BTA 4 identified by Sbardella et al. (2021) was found to be similar with the genomic window located between 42.2 and 43.4 Mb of BTA 4 identified in our study (Sbardella et al., 2021). Moreover, one of the candidate genes highlighted in our study, *ARHGAP18,* located within the window 67.8–69.2 Mb of BTA9, is a protective gene known to maintain endothelial cell alignments. The deletion of *ARHGAP18* could lead to a loss of endothelial cells, which could affect the overall health and longevity of the animal (Lay et al., 2019). Candidate gene *KCNC1*, annotated within the window 34.5–34.9 Mb of BTA15, is a member of the family of integral proteins that mediate the control of the flow of potassium ions across biological membranes, thereby regulating membrane excitability (Alam et al., 2023). Potassium is an essential nutrient for animals, important for muscle contraction and normal cardiac function, especially in ensuring the longevity of animals (Dabbir and Rajavolu, 2024). Other candidate genes highlighted for stayability are associated with lactation (GO:0007595), female pregnancy (GO:0007565), mammary gland development (GO:0030879), regulation of secretion (GO:0051046), nucleic acid binding (GO:0003676), Prolactin signaling pathway (KEGG:04,917) and DNA binding (GO:0003677). These genomic regions contribute to the phenotypic expression of stayability by encoding genes that regulate essential physiological processes such as endothelial cell maintenance, ion transport, and lactation, which are essential for enhancing cow longevity, productivity and overall herd profitability.

#### 4.2.12 Weights at 120, 210, 365 and 450 days

Body weight is a common criterion considered in beef cattle production, as it is easily measured and responds well to selection due to its moderate to high heritability estimates (Yokoo et al., 2007; Boligon et al., 2009). In this study, standardized weight records were taken at 120, 210, 365 and 450 days of age (W120, W210, W365, and W450, respectively). The top 1% genomic windows, associated with weight at various ages, were distributed across multiple chromosomes. For weight at 120 days, these windows spanned 12 chromosomes (BTA3, BTA5, BTA6, BTA7, BTA8, BTA10, BTA11, BTA14, BTA16, BTA19, BTA20, BTA28) and explained 2.995% of the additive genetic variance, coding 373 genes (Table 13, Supplementary Table S13). At 210 days, the top 1% genomic windows spanned 14 chromosomes (BTA3, BTA5, BTA6, BTA7, BTA8, BTA10, BTA11, BTA14, BTA16, BTA19, BTA20, BTA21, BTA23, BTA28) and explained 2.980% of the additive genetic variance, coding 306 genes (Table 14., Supplementary Table S14). For weight at 365 days, the top 1% genomic windows were found across 14 chromosomes (BTA3, BTA5, BTA6, BTA8, BTA10, BTA11, BTA14, BTA16, BTA19, BTA20, BTA21, BTA23, BTA27, BTA28), and explained 2.869% of the additive genetic variance, coding for 352 genes (Table 15., Supplementary Table S15A). Finally, for weight at 450 days, the top 1% genomic windows were found in 15 chromosomes (BTA1, BTA2, BTA3, BTA5, BTA6, BTA7, BTA10, BTA11, BTA13, BTA14, BTA15, BTA16, BTA19, BTA20, BTA21, BTA24, BTA28) and explained 2.867% of the additive genetic variance, coding 353 genes (Table 16., Supplementary Table S15B)

**TABLE 13:** Candidate genes located within the top 1% genomic windows associated with weight at 120 days of age in Nellore cattle.

BTA	Position (BP)	Candidate genes	^1^Var (%)
10	82,912,907–83,689,406	*SIPA1L1, RGS6*	0.218
5	39,340,288–40373,980	*PDZRN4, CNTN1*	0.191
6	69,176,844–69,912,628	*CHIC2, GSX2,PDGFRA*	0.177
20	29,800,340–31,105,010	*MRPS30, FGF10*	0.175
11	29,718,581–30428,257	*EPCAM, MSH2,KCNK12,MSH6,FBXO11*	0.162
19	40,595,675–42,117,748	*TOP2A, IGFBP4, TNS4, KRT222, CCR7, KRT27, KRT24, KRT10, JUP, P3H4, FKBP10, NT5C3B, KLHL10, ACLY, ODAD4, KRT12, KRT20, KRT23, KRT39, KRT40, HAP1, GAST, KRT14, KRT9, KRT16, GJD3, EIF1, KRTAP4-7, KRTAP9-2, KRTAP16-1, KRT31, KRT37, KRT36, KRTAP3-3, KRTAP3-1, KRTAP17-1, SMARCE1, KRT28, KRT25, KLHL11, KRT17, KRT42, KRT19, KRT15, KRT33A, KRT32*	0.157
8	67,230,723–68,017,522	*SLC18A1, ATP6V1B2, LZTS1*	0.156
10	85,365,431–86,375,387	*ENTPD5, BBOF1, ALDH6A1, LIN52, VSX2, ABCD4, VRTN, SYNDIG1L, NPC2, ISCA2, LTBP2, AREL1, FCF1, YLPM1, PROX2, DLST, RPS6KL1, PGF, EIF2B2, ACYP1, ZC2HC1C, NEK9, TMED10, MLH3*	0.154
8	25,904,409–27,055,794	*ADAMTSL1, SH3GL2, CNTLN*	0.149
5	40,614,933–41,734,874	*SLC2A13, C12orf40,ABCD2,KIF21A,LRRK2*	0.143
16	60,666,257–61,711,110	*SOAT1, AXDND1, NPHS2, TDRD5, FAM163A, TOR1AIP2, TOR1AIP1, CEP350, QSOX1, LHX4, ACBD6*	0.143
7	66,369,626–67,211,667	*-*	0.135
28	43,952,431–45,154,855	*MARCHF8, ALOX5, ZNF22, DEPP1, RASSF4, TMEM72, CXCL12, PARG, TIMM23, NCOA4, MSMB, FAM21A, ZFAND4, OR13A1, OR6D16*	0.134
19	37,595,473–39,850,336	*SKAP1, SNX11, CBX1, NFE2L1, COPZ2, LASP1, CDK5RAP3, FBXO47, PRR15L, PNPO, SP2, SNF8, UBE2Z, ATP5MC1, SPMAP1, RPL23, HOXB9, HOXB8, HOXB7, SP6, SCRN2, LRRC46, MRPL10, OSBPL7, CALCOCO2, TTLL6, HOXB4, HOXB5, HOXB3, HOXB2, HOXB1, HOXB13, PLXDC1, CACNB1, RPL19, STAC2, FBXL20, MED1, MLLT6, ARHGAP23, SRCIN1, EPOP, PCGF2, PSMB3, PIP4K2B, CWC25, TBKBP1, KPNB1, NPEPPS, MRPL45, GPR179, SOCS7, TBX21, LASP1NB, HOXB6*	0.133
14	10,584,341–11,176,477	*GSDMC, CYRIB, ASAP1*	0.133
3	32,282,358–33,320,159	*CD53, KCNA3, LAMTOR5, KCNA2, KCNA10, CYM, SLC16A4, RBM15, PROK1, DRAM2, LRIF1, KCNC4, SLC6A17, UBL4B*	0.132
14	21,415,611–22,184,329	*TCEA1, LYPLA1, MRPL15, OPRK1, RB1CC1, RGS20, ATP6V1H, NPBWR1*	0.127
14	41,527,200–42,563,372	*PKIA, IL7*	0.127
14	4,009,175–4550,266	*COL22A1, FAM135B*	0.126
8	8,0,792,014–81,428,622	*DAPK1, CTSL, FBP2, AOPEP, FBP1*	0.125

Proportion of the trait variance explained by each window (%).

**TABLE 14:** Candidate genes located within the top 1% genomic windows associated with weight at 210 days of age in Nellore cattle.

BTA	Position (BP)	Candidate genes	^1^Var (%)
10	82,912,907–83,689,406	*SIPA1L1, RGS6*	0.212
6	69,176,844–69,912,628	*CHIC2, GSX2, PDGFRA*	0.185
5	39,325,569–40371,388	*PDZRN4, CNTN1*	0.176
10	85,381,223–86,410,610	*BBOF1, ALDH6A1, LIN52, VSX2, ABCD4, VRTN, SYNDIG1L, NPC2, ISCA2, LTBP2, AREL1, FCF1, YLPM1, PROX2, DLST, RPS6KL1, PGF, EIF2B2, ACYP1, ZC2HC1C, NEK9, TMED10, MLH3*	0.170
20	29,800,340–31,105,010	*MRPS30, FGF10*	0.169
16	60,618,654–61,579,318	*SOAT1, AXDND1, NPHS2, TDRD5, FAM163A, TOR1AIP2, TOR1AIP1, CEP350, QSOX1, LHX4, ACBD6*	0.165
11	29,718,581–30428,257	*EPCAM, MSH2, KCNK12, MSH6,FBXO11*	0.152
5	40,614,933–41,734,874	*SLC2A13, C12orf40, ABCD2, KIF21A,LRRK2*	0.148
8	67,230,723–68,017,522	*SLC18A1, ATP6V1B2, LZTS1*	0.148
14	41,527,200–42,563,372	*PKIA, IL7*	0.138
14	10,584,341–11,176,477	*GSDMC, CYRIB, ASAP1*	0.137
3	32,225,556–33,265,065	*CD53, KCNA3, LAMTOR5, KCNA2, KCNA10, CYM, SLC16A4, RBM15, PROK1, CEPT1, DRAM2, LRIF1, KCNC4, SLC6A17*	0.136
19	40,615,001–42,124,668	*TOP2A, IGFBP4, TNS4, KRT222, CCR7, KRT27, KRT24, KRT10, JUP, P3H4, FKBP10, NT5C3B, KLHL10, ACLY, ODAD4, KRT12, KRT20, KRT23, KRT39, KRT40, HAP1, GAST, KRT14, KRT9, KRT16, EIF1, KRTAP4-7, KRTAP9-2, KRTAP16-1, KRT31, KRT37, KRT36, KRTAP3-3, KRTAP3-1, KRTAP17-1, SMARCE1, KRT28, KRT25, KLHL11, KRT17, KRT42, KRT19, KRT15, KRT33A, KRT32*	0.135
8	25,904,409–27,055,794	*ADAMTSL1, SH3GL2, CNTLN*	0.135
28	8,749,119–9703,912	*EDARADD, LGALS8, HEATR1, ACTN2, MTR, NID1, RYR2, GPR137B, ERO1B*	0.132
21	35,060,169–35,613,940	*STXBP6*	0.131
23	12,674,482–13,935,150	*DNAH8, GLP1R, SAYSD1, KCNK5, KCNK17, KCNK16, KIF6, DAAM2, MOCS1*	0.130
7	66,369,626–67,211,667	*-*	0.128
14	4,009,175–4550,266	*COL22A1, FAM135B*	0.127
28	43,952,431–45,154,855	*MARCHF8, ALOX5, ZNF22, DEPP1, RASSF4, TMEM72, CXCL12, PARG, TIMM23, NCOA4, MSMB, FAM21A, ZFAND4, OR13A1, OR6D16*	0.127

Proportion of the trait variance explained by each window (%).

**TABLE 15:** Candidate genes located within the top 1% genomic windows associated with weight at 365 days of age in Nellore cattle.

BTA	Position (BP)	Candidate genes	^1^Var (%)
6	69,245,821–70037493	*CHIC2,GSX2,PDGFRA*	0.190
16	60,596,486–61,534,166	*SOAT1, AXDND1, NPHS2, TDRD5, FAM163A, TOR1AIP2, TOR1AIP1, CEP350, QSOX1, LHX4, ACBD6*	0.189
10	82,912,907–83,689,406	*SIPA1L1, RGS6*	0.181
10	85,381,223–86,410,610	*BBOF1, ALDH6A1, LIN52, VSX2, ABCD4, VRTN, SYNDIG1L, NPC2, ISCA2, LTBP2, AREL1, FCF1, YLPM1, PROX2, DLST, RPS6KL1, PGF, EIF2B2, ACYP1, ZC2HC1C, NEK9, TMED10, MLH3*	0.156
20	29,800,340–31,105,010	*MRPS30, FGF10*	0.152
5	39,325,569–40371,388	*PDZRN4, CNTN1*	0.150
11	29,718,581–30428,257	*EPCAM, MSH2, KCNK12, MSH6, FBXO11*	0.145
28	8,749,119–9703,912	*EDARADD, LGALS8, HEATR1, ACTN2, MTR, NID1, RYR2, GPR137B, ERO1B*	0.142
14	41,537,492–42,576,327	*PKIA, IL7*	0.140
14	8,999,569–9960,007	*KCNQ3, HHLA1, OC90, EFR3A, ADCY8*	0.135
8	25,904,409–27,055,794	*ADAMTSL1, SH3GL2, CNTLN*	0.134
8	67,230,723–68,017,522	*SLC18A1, ATP6V1B2, LZTS1*	0.134
27	39,570,348–40785,408	*OXSM, NGLY1, TOP2B, RARB*	0.130
19	41,383,415–43,525,430	*COASY, MLX, PSMC3IP, RETREG3, TUBG2, PLEKHH3, CCR10, EZH1, RAMP2, VPS25, WNK4, COA3, CNTD1, BECN1, PSME3, AOC2, AOC3, G6PC1, JUP, P3H4, FKBP10, NT5C3B, KLHL10, ACLY, ODAD4, CNP, DNAJC7, NKIRAS2, ZNF385C, DHX58, KAT2A, HSPB9, RAB5C, KCNH4, HCRT, GHDC, STAT5B, STAT5A, AARSD1, AARSD1, RUNDC1, RPL27, STAT3, IFI35, VAT1, RND2, BRCA1, NBR1, TMEM106A, CAVIN1, ATP6V0A1, NAGLU, HSD17B1, ARL4D, DHX8, ETV4, HAP1, GAST, KRT14, KRT9, KRT16, EIF1, KRTAP9-2, KRTAP16-1, KRT31, KRT37, KRT36, KRTAP17-1, TUBG1, CNTNAP1, KLHL11, KRT17, KRT42, KRT19, KRT15, KRT33A, KRT32*	0.129
23	11,607,995–13,214,326	*ZFAND3, BTBD9, GLO1, DNAH8, GLP1R, SAYSD1, KCNK5, KCNK17, KCNK16, KIF6, MDGA1*	0.129
3	95,983,147–97,150,304	*ELAVL4*	0.128
3	32,225,556–33,265,065	*CD53, KCNA3, LAMTOR5, KCNA2, KCNA10, CYM, SLC16A4, RBM15, PROK1, CEPT1, DRAM2, LRIF1, KCNC4, SLC6A17*	0.128
21	35,060,169–35,613,940	*STXBP6*	0.127
14	10,584,341–11,176,477	*GSDMC, CYRIB, ASAP1*	0.126
8	8,0,792,014–81,428,622	*DAPK1, CTSL, FBP2,AOPEP,FBP1*	0.124

Proportion of the trait variance explained by each window (%).

**TABLE 16:** Candidate genes located within the top 1% genomic windows associated with weight at 450 days of age in Nellore cattle.

BTA	Position (BP)	Candidate genes	^1^Var (%)
6	69,245,821–70037493	*CHIC2, GSX2, PDGFRA*	0.196
16	60,596,486–61,534,166	*SOAT1, AXDND1, NPHS2, TDRD5, FAM163A, TOR1AIP2, TOR1AIP1, CEP350, QSOX1, LHX4, ACBD6*	0.194
10	82,998,446–83,815,442	*SIPA1L1, RGS6*	0.183
10	85,365,431–86,375,387	*ENTPD5, BBOF1, ALDH6A1, LIN52, VSX2, ABCD4, VRTN, SYNDIG1L, NPC2, ISCA2, LTBP2, AREL1, FCF1, YLPM1, PROX2, DLST, RPS6KL1, PGF, EIF2B2, ACYP1, ZC2HC1C, NEK9, TMED10, MLH3*	0.176
20	29,800,340–31,105,010	*MRPS30, FGF10*	0.145
11	29,718,581–30428,257	*EPCAM, MSH2, KCNK12, MSH6, FBXO11*	0.140
28	8,749,119–9703,912	*EDARADD, LGALS8, HEATR1, ACTN2, MTR, NID1, RYR2, GPR137B, ERO1B*	0.140
5	39,325,569–40371,388	*PDZRN4, CNTN1*	0.140
8	25,904,409–27,055,794	*ADAMTSL1, SH3GL2, CNTLN*	0.135
23	12,674,482–13,935,150	*DNAH8, GLP1R, SAYSD1, KCNK5, KCNK17, KCNK16, KIF6, DAAM2, MOCS1*	0.133
14	41,537,492–42,576,327	*PKIA, IL7*	0.133
14	10,584,341–11,176,477	*SDMC, CYRIB, ASAP1*	0.132
19	41,383,415–43,525,430	*COASY, MLX, PSMC3IP, RETREG3, TUBG2, PLEKHH3, CCR10, EZH1, RAMP2, VPS25, WNK4, COA3, CNTD1, BECN1, PSME3, AOC2, AOC3, G6PC1, JUP, P3H4, FKBP10, NT5C3B, KLHL10, ACLY, ODAD4, CNP, DNAJC7, NKIRAS2, ZNF385C, DHX58, KAT2A, HSPB9, RAB5C, KCNH4, HCRT, GHDC, STAT5B, STAT5A, AARSD1, AARSD1, RUNDC1, RPL27, STAT3, IFI35, VAT1, RND2, BRCA1, NBR1, TMEM106A, CAVIN1, ATP6V0A1, NAGLU, HSD17B1, ARL4D, DHX8, ETV4, HAP1, GAST, KRT14, KRT9, KRT16, EIF1, KRTAP9-2, KRTAP16-1, KRT31, KRT37, KRT36,KRTAP17-1, TUBG1, CNTNAP1, KLHL11, KRT17, KRT42, KRT19, KRT15, KRT33A, KRT32*	0.130
3	32,225,556–33,265,065	*CD53, KCNA3, LAMTOR5, KCNA2, KCNA10, CYM, SLC16A4, RBM15, PROK1, CEPT1, DRAM2, LRIF1, KCNC4, SLC6A17*	0.128
5	40,614,933–41,734,874	*SLC2A13, C12orf40, ABCD2, KIF21A, LRRK2*	0.128
28	38,562,781-39439768	*GHITM, GPR15LG, CDHR1, LRIT2, LRIT1, RGR, CCSER2*	0.128
14	8,999,569-9960007	*KCNQ3, HHLA1, OC90, EFR3A, ADCY8*	0.127
27	39,570,348-40785408	*OXSM, NGLY1, TOP2B, RARB*	0.127
9	80,031,480-80701610	*HIVEP2, AIG1*	0.126
21	35,060,169-35613940	*STXBP6*	0.125

Proportion of the trait variance explained by each window (%).

It is noteworthy that similar genomic windows were observed across weights taken at different ages. For instance, all genomic windows identified for W120 were also identified at the other ages. Interestingly, the windows found for W210 also included the ones reported in BTA21 and BTA23, in addition to the ones observed for W120. Similarly, for W365, a genomic region on BTA27 was observed together with the regions annotated for W210 (i.e., all identified for W120 plus the ones located in BTA21, BTA23, and BTA27). For W450, the genomic regions identified in BTA21, BTA24, and BTA28 were added to the regions also annotated for W120. As the proportion of the total variance explained by the top 1% windows remains similar across the ages, this finding suggests that weight taken at older ages can be even more polygenic than weights taken at young ages.

The candidate gene *DAAM2*, located in the window 12.6–13.9 Mb on BTA23, is known to enable actin-binding activity and regulation of the Wnt signaling pathway, which is required for various processes during development (Alliance of Genome Resources, April 2022). The candidate gene *NGLY*1, located in window 39.5–40.7 Mb of BTA27, is a gene that plays a key role in the proteasome-mediated degradation of misfolded glycoproteins and has been shown to affect the development and physiology of animals in cases of deficiency (Pandey et al., 2022). The candidate gene *TOP2B*, located in the window 39.5–40.7 Mb of BTA27 is essential in vertebrate development (Austin et al., 2018).

Other candidate genes highlighted for weights at different ages are related to intermediate filament organization (GO:0000245), potassium ion transport (GO:0000377), supramolecular fiber organization GO:0006397, epithelial cell differentiation (GO:0007010), epithelium development (GO:0006996), organelle organization (GO:0070672), cell body membrane GO:0008076, determination of adult lifespan (GO:0005686), and cellular component organization (GO:0006813). These genomic regions contribute to the phenotypic expression of body weight across different ages by encoding genes that regulate biological processes such as actin-binding, ion transport and cellular organization, all of which are essential for skeletal growth, muscle development and overall body mass accumulation in cattle.

## 5 Conclusion

This study is the first GWAS using over 300k genotyped animals from the Nellore population. We used the APY to identify the top 1% genomic windows for each trait and annotated the positional candidate genes and genomic regions within them. The traits included in this study are currently evaluated by ABCZ. The top 1% windows for all traits explained between 2.779% (stayability) to 3.158% additive genetic variance (finishing score), underscoring the polygenic characteristic of these traits. The functional analysis of the candidate genes and genomic regions revealed several functionally significant genes that enhance our understanding of the genetic architecture underlying these important traits in Nellore cattle. Notably, some identified regions have been previously reported in the literature, while others are novel discoveries that warrant further investigation. These findings may facilitate gene prioritization efforts, helping to identify functional candidate genes that can enhance genomic selection strategies for economically important traits in Nellore cattle. Ultimately, this research lays a foundation for future studies aimed at improving genetic selection and advancing the productivity and profitability of Nellore cattle breeding programs.

## Data Availability

The raw data supporting the conclusions of this article will be made available by the authors, without undue reservation.
